# Usual blood pressure, peripheral arterial disease, and vascular risk: cohort study of 4.2 million adults

**DOI:** 10.1136/bmj.h4865

**Published:** 2015-09-29

**Authors:** Connor A Emdin, Simon G Anderson, Thomas Callender, Nathalie Conrad, Gholamreza Salimi-Khorshidi, Hamid Mohseni, Mark Woodward, Kazem Rahimi

**Affiliations:** 1 The George Institute for Global Health, Oxford Martin School, University of Oxford, Oxford OX1 3DB, UK; 2 The George Institute for Global Health, University of Sydney, Sydney, Australia; 3 Department of Epidemiology, Johns Hopkins University, Baltimore, MD, USA; 4 Division of Cardiovascular Medicine, Radcliffe Department of Medicine, University of Oxford, Oxford, UK

## Abstract

**Objectives** To determine the subgroup specific associations between usual blood pressure and risk of peripheral arterial disease, and to examine the relation between peripheral arterial disease and a range of other types of vascular disease in a large contemporary cohort.

**Design** Cohort study.

**Setting** Linked electronic health records from 1990 to 2013 in the United Kingdom.

**Participants** 4 222 459 people aged 30-90 years, registered at a primary care practice for at least one year and with a blood pressure measurement.

**Main outcome measures** Time to first diagnosis of new onset peripheral arterial disease and time to first diagnosis of 12 different vascular events.

**Results** A 20 mm Hg higher than usual systolic blood pressure was associated with a 63% higher risk of peripheral arterial disease (hazard ratio 1.63, 95% confidence interval 1.59 to 1.66). The strength of the association declined with increasing age and body mass index (P<0.001 for interaction) but was not modified by sex or smoking status. Peripheral arterial disease was associated with an increased risk of 11 different vascular events, including ischaemic heart disease (1.68, 1.58 to 1.79), heart failure (1.63, 1.52 to 1.75), aortic aneurysm (2.10, 1.79 to 2.45), and chronic kidney disease (1.31, 1.25 to 1.38), but not haemorrhagic stroke. The most common initial vascular event among those with peripheral arterial disease was chronic kidney disease (24.4% of initial events), followed by ischaemic heart disease (18.5% of initial events), heart failure (14.7%), and atrial fibrillation (13.2%). Overall estimates from this cohort were consistent with those derived from traditional studies when we pooled the findings in two meta-analyses.

**Conclusions** Raised blood pressure is a strong risk factor for peripheral arterial disease in a range of patient subgroups. Furthermore, clinicians should be aware that those with established peripheral arterial disease are at an increased risk of a range of other vascular events, including chronic kidney disease, ischaemic heart disease, heart failure, atrial fibrillation, and stroke.

## Introduction

In 2010 an estimated 202 million people globally had peripheral arterial disease.^1^ The prevalence of the disease increases with age, from 1% of the population at age 40-49 years to 22.4% at age 80 or older.^2^ In an analysis of 48 294 study participants, peripheral arterial disease was associated with twice the risk of all cause mortality and three times the risk of death from vascular causes over a mean follow-up of 10 years.^3^ Despite widespread availability and the use of effective risk modifying interventions in the United States^4^ and Europe,^5^ peripheral arterial disease remains a leading cardiovascular cause of morbidity and mortality, and worldwide the burden from the disease is increasing.^6^

Raised blood pressure is a known risk factor for peripheral arterial disease.^7^ ^8^ Previously reported associations have, however, varied substantially, possibly as a result of the small size of existing prospective studies^9^ ^10^ or lack of adequate adjustment for time dependent regression dilution bias^11^ (see supplementary table 1 and supplementary figure 1) Furthermore, the strength of the association of blood pressure with incident peripheral arterial disease by patient characteristics (for example, smoking status and body mass index) is unclear.

Peripheral arterial disease is a further risk factor for vascular disease, particularly coronary heart disease and stroke. In the meta-analysis by the Ankle Brachial Index Collaboration, peripheral arterial disease was associated with a 96% higher risk of fatal cardiovascular disease, 45% higher risk of coronary heart disease, and 35% higher risk of stroke.^12^ However, the relation between peripheral arterial disease and other vascular outcomes, including chronic kidney disease, atrial fibrillation, thromboembolism, and stroke subtypes, is unclear. Reliable quantification of the vascular risk associated with peripheral arterial disease, in a contemporary setting, is necessary for more targeted approaches to prevention and management.

We conducted an analysis of 4 222 459 participants in a contemporary setting using validated and linked electronic health records. As a complementary approach, we compared our results to meta-analyses of prospective studies.

## Methods

### Participants and exposures

We used prospectively collected electronic health records, previously validated and used for epidemiological research,^11^ ^13^ to identify a cohort of 4.2 million people. Electronic health records were combined from Clinical Practice Research Datalink (primary care) and, for eligible participants, Hospital Episode Statistics (secondary care), and the Office for National Statistics (cause specific mortality). We included all those aged between 30 and 90 years with no history of cardiovascular disease (with the exception of peripheral arterial disease) and a blood pressure measurement taken at a research standard practice (a practice meeting certain research quality standards) between 1990 and 2013. The exposure was this first blood pressure measurement. Covariates, including age, sex, body mass index, smoking status, and total cholesterol and high density lipoprotein cholesterol levels were taken as the closest recorded measurement within two years of the baseline blood pressure measurement. Patients were defined as having baseline antihypertensive treatment (*British National Formulary* chapters 2.2.1, 2.2.3-5 and 2.6.2) or lipid lowering treatment (chapter 2.12) if they were prescribed an antihypertensive or lipid lowering drug in the two years before a baseline blood pressure measurement. We classified people as having peripheral arterial disease at baseline if they had a previous diagnosis (in primary care or secondary care) of peripheral arterial disease before their blood pressure measurement. To identify covariates and endpoints, we used publicly available Read codes from the clinical codes repository.^14^

### Endpoints

For the analysis of the relation between blood pressure and risk of peripheral arterial disease, the primary endpoint was the first diagnosis of peripheral arterial disease (see supplementary table 2). Participants were censored at the first diagnosis of peripheral arterial disease (either in primary care, in secondary care, or by mortality), transfer out of practice, death, or last collection date of practice.

For the analysis of the relation between peripheral arterial disease and vascular event, the primary endpoint was the first of 12 different vascular events: ischaemic heart disease, heart failure, atrial fibrillation, valvular heart disease, chronic kidney disease, ischaemic stroke, hemorrhagic stroke, unspecified stroke, aortic aneurysm, deep vein thrombosis, pulmonary embolism, and vascular dementia (see supplementary table 2). Participants were censored at the first of any of the 12 different vascular events, transfer out of practice, death, or last collection date of practice.

### Statistical analysis

Cox models, stratified by practice to account for clustering at the practice level, examined the relation between blood pressure and incident peripheral arterial disease, and the relation between baseline peripheral arterial disease and incident vascular disease. We excluded those with pre-existing peripheral arterial disease from the analysis of blood pressure and incident peripheral arterial disease. Blood pressure was analysed both as a continuous variable (for each 20 mm Hg higher systolic blood pressure and 10 mm Hg higher diastolic blood pressure) and as a categorical variable. The primary model was adjusted for age, sex, body mass index, diabetes, antihypertensive use, and lipid lowering drug use.

Measurement error in a blood pressure measurement and fluctuations in blood pressure will bias any association between measured blood pressure and an outcome of the interest to the null. To correct for this regression dilution bias,^15^ we used usual blood pressure as our exposure, applying similar methods to those of the Emerging Risk Factors Collaboration.^16^ We used generalised estimating equations to regress serial blood pressure measurements within the median follow-up of this study on the baseline blood pressure measurement. For systolic blood pressure and diastolic blood pressures, we calculated regression dilution ratios of 2.2 and 2.6, respectively. Blood pressure was analysed both as a continuous variable (for each 20 mm Hg and 10 mm Hg higher blood pressure) and as a categorical variable. We defined usual systolic blood pressure categories by the measured systolic blood pressure categories: <105, 106-115, 116-125, 126-135, 136-145, 146-155, 156-165, 166-175, 176-185, 186-195, and >195 mm Hg. Usual diastolic blood pressure categories were defined by the measured diastolic blood pressure categories: <65, 66-75, 76-85, 86-95, 96-105, 106-115, >115 mm Hg. We plotted hazard ratios against the mean of each blood pressure category. Age at baseline was analysed both as a continuous variable and as a categorical variable, defined by the following categories: 30-40, 41-50, 51-60, 61-70, 71-90 years.

Multiple imputation using chained equations was used to impute missing covariates; five imputations were generated.

To estimate the absolute increase in risk of peripheral arterial disease associated with a 20 mm Hg higher systolic blood pressure and a 10 mm Hg higher diastolic blood pressure in our cohort, we multiplied the absolute incidence of peripheral arterial disease in our cohort (in events per 100 000 person years) by the adjusted hazard ratio associated with a 20 mm Hg higher than usual systolic blood pressure and a 10 mm Hg higher than usual diastolic blood pressure. To estimate the absolute increase in risk of cardiovascular disease associated with baseline peripheral arterial disease in our cohort, we multiplied the incidence of cardiovascular disease in the population without peripheral arterial disease by the adjusted hazard ratio of cardiovascular disease associated with peripheral arterial disease.

### Sensitivity analyses

Eight sensitivity analyses were conducted. Firstly, we further adjusted models for total and high density lipoprotein cholesterol levels. Secondly, to account for potential cohort effects we further adjusted models for period of blood pressure measurement. Thirdly and fourthly, to examine whether results were influenced by reverse causality we excluded the first two and four years of follow-up. Fifthly, for the analysis of baseline blood pressure and incident peripheral arterial disease we excluded those receiving antihypertensive treatments at baseline. Sixthly, for the analysis of baseline peripheral arterial disease and incident vascular disease we excluded those with a diagnosis of peripheral arterial disease more than three years before, to restrict cases to recent diagnoses of peripheral arterial disease. Seventhly, from the analysis of baseline peripheral arterial disease and incident vascular disease we excluded all those who developed peripheral arterial disease during follow-up but did not have peripheral arterial disease at baseline. Finally, for the association between blood pressure and incident peripheral arterial disease and baseline peripheral arterial disease and incident cardiovascular disease, we examined only fatal cardiovascular events.

### Meta-analyses

Two meta-analyses were performed: for blood pressure related to incident peripheral arterial disease and for baseline peripheral arterial disease related to vascular disease. For the blood pressure meta-analysis, an experienced research librarian designed a search strategy to identify previous studies on the association of blood pressure and peripheral arterial disease, published on Medline between January 1996 and February 2015. No language restrictions were applied. We considered studies to be eligible if they were prospective and had 100 or more participants, had a median follow-up of at least six months, and reported an association between blood pressure and incident peripheral arterial disease that could be standardised for each 20 mm Hg higher systolic blood pressure. Only cohort studies were included; we excluded cross sectional and case-control studies. Measures extracted in duplicate were relative risks (for example, hazard ratios, odds ratios), population size, characteristics of the study population, number of incident peripheral arterial disease events, and adjustments. Two studies used an average of repeated blood pressure measurements to attempt to control for regression dilution bias. However, four studies did not attempt to adjust for regression dilution bias by using multiple blood pressure measurements; we applied our regression dilution ratio of 2.2 to these studies before meta-analysis. One study reported a relative risk for each 1 mm Hg higher diastolic blood pressure, and we assumed that this corresponded to a 2 mm Hg higher systolic blood pressure.^17^ From previous cohort studies with our estimate we used random effects meta-analysis to pool relative risks, standardised for each 20 mm Hg higher systolic blood pressure. We used the I^2^ statistic to quantify heterogeneity between studies.

For the meta-analysis of baseline peripheral arterial disease and incident vascular disease, we pooled the relative risks for vascular death, ischaemic heart disease, and stroke from our cohort with those reported by the Ankle Brachial Index Collaboration meta-analysis.^12^ We used random effects meta-analysis to pool relative risks, and quantified heterogeneity between studies using the I^2^ statistic. Analyses were conducted using R version 3.0.

### Patient involvement

No patients were involved in setting the research question or the outcome measures, nor were they involved in the design and implementation of the study. There are no plans to involve patients in dissemination.

## Results

After excluding 11 014 472 ineligible individuals based on the inclusion criteria, 4 222 459 people qualified for our analysis (see supplementary figure 2). Of these potential participants, 18 269 (0.43%) had peripheral arterial disease at baseline and were excluded from the analysis of blood pressure and risk of peripheral arterial disease. The median age was 46 (interquartile range 36-59) years, and the median body mass index was 25.8 (23.0-29.3, table 1). Of the 4 204 190 participants without peripheral arterial disease at baseline, 44 329 (1.05%) developed peripheral arterial disease during follow-up and 485 760 (11.55%) had a non-peripheral arterial disease vascular event over a median follow-up of 7.0 years. Of the 18 269 participants with peripheral arterial disease at baseline, 7760 (42.5%) had a non-peripheral arterial disease vascular event during follow-up. See supplementary table 3 for the proportion of different ICD codes for hospital admission for peripheral arterial disease at different baseline ages.

**Table 1 tbl1:** Characteristics of participants. Values are numbers (percentages) of participants unless stated otherwise

Characteristics	Peripheral arterial disease at baseline		Overall
	Present	Absent	
No of participants	18 269	4 204 190	4 222 459
Median (interquartile range) age at baseline	69 (61-77)	46 (36-59)	46 (36-59)
Women	7679 (42.0)	2 333 871 (55.5)	2 341 550 (55.5)
Median (interquartile range) body mass index	25.6 (22.8-28.9)	25.8 (23.0-29.3)	25.8 (23.0-29.3)
Median (interquartile range) blood pressure (mm Hg)	147 (130-160)	130 (120-144)	130 (120-144)
Smoking status:			
Current smoker	6218 (44.0)	957 298 (28.0)	963 516 (28.1)
Never smoker	3864 (27.3)	1 856 810 (54.3)	1 860 674 (54.2)
Former smoker	4061 (28.7)	604 968 (17.7)	609 029 (17.7)
Median (interquartile range) cholesterol level:			
Total	5.3 (4.5-6.2)	5.5 (4.7-6.2)	5.5 (4.7-6.2)
High density lipoprotein	1.3 (1.1-1.6)	1.4 (1.1-1.6)	1.4 (1.1-1.6)
Most deprived fifth*	4101 (22.4)	795 665 (18.9)	799 766 (18.9)
Antihypertensive treatment:			
At baseline	6212 (34.0)	417 900 (9.9)	424 112 (10.0)
During follow-up	11 384 (62.3)	1 207 609 (28.7)	1 218 993 (28.9)
Lipid lowering treatment:			
At baseline	2528 (13.8)	79 261 (1.9)	81 789 (1.9)
During follow-up	7946 (43.5)	650 560 (15.5)	658 506 (15.6)
Diabetes at baseline	2690 (14.7)	133 878 (3.2)	136 568 (3.2)

Proportion of patients with missing covariates: body mass index (30.5%), smoking (18.7%), total cholesterol (73.0%), high density lipoprotein cholesterol (80.2%).

*Lowest fifth of socioeconomic status.

### Association of usual blood pressure with risk of peripheral arterial disease

No evidence of a nadir in the relation between usual systolic blood pressure and incident peripheral arterial disease was observed in the range 115-170 mm Hg (fig 1). The strength of the association declined with increasing age, from a hazard ratio of 2.51 for each 20 mm Hg higher than usual systolic blood pressure at age 30-40, to 1.36 (95% confidence interval 1.31 to 1.41) at age 71-90 (figs 1 and 2). The association between 20 mm Hg systolic blood pressure and risk of peripheral arterial disease also declined with increasing body mass index, from a hazard ratio of 1.72 at body mass index ≤25 to 1.35 at body mass index ≥35 (fig 2). However, the absolute risk difference associated with a 20 mm Hg higher than usual systolic blood pressure increased with increasing body mass index and age, owing to the higher baseline absolute risk of peripheral arterial disease. Tests for interaction by smoking status and sex were not significant. Overall, a 20 mm Hg higher than usual systolic blood pressure was associated with a 63% higher risk of peripheral arterial disease (hazard ratio 1.63, 95% confidence interval 1.59 to 1.66) and a 10 mm Hg higher than usual diastolic blood pressure was associated with a 35% higher risk of peripheral arterial disease (1.35, 1.31 to 1.38, see supplementary fig 3). Supplementary table 4 shows the risk of peripheral arterial disease for different units of blood pressure. One standard deviation of systolic blood pressure was associated with a significantly greater risk of peripheral arterial disease than one standard deviation of diastolic blood pressure, indicating that systolic blood pressure is more strongly associated with risk of peripheral arterial disease than is diastolic blood pressure (supplementary table 4). Estimates were similar in various sensitivity analyses (see supplementary figures 4-9 and supplementary table 5). Corresponding absolute increases in risk of peripheral arterial disease associated with a 20 mm Hg higher systolic blood pressure and a 10 mm Hg higher diastolic blood pressure were 85 events and 47 events per 100 000 person years of follow-up, respectively.

**Figure fig1:**
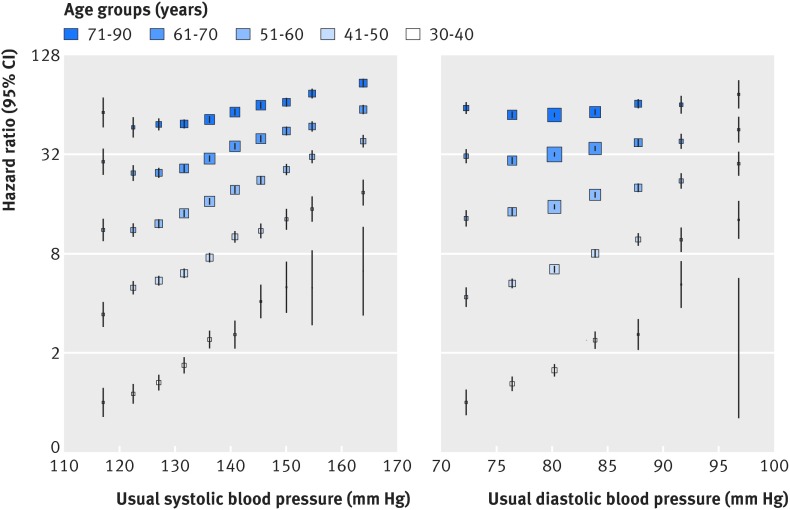
**Fig 1** Adjusted hazard ratios of systolic blood pressure and diastolic blood pressure for incident peripheral arterial disease by age. Adjustments were for body mass index, smoking status, sex, baseline diabetes, and baseline antihypertensive and lipid lowering drug use, and the interaction between age as a categorical variable and systolic and diastolic blood pressures as categorical variables, respectively. Confidence intervals are displayed as floating absolute risks. Hazard ratios are plotted at the mean of each usual systolic blood pressure and usual diastolic blood pressure category

**Figure fig2:**
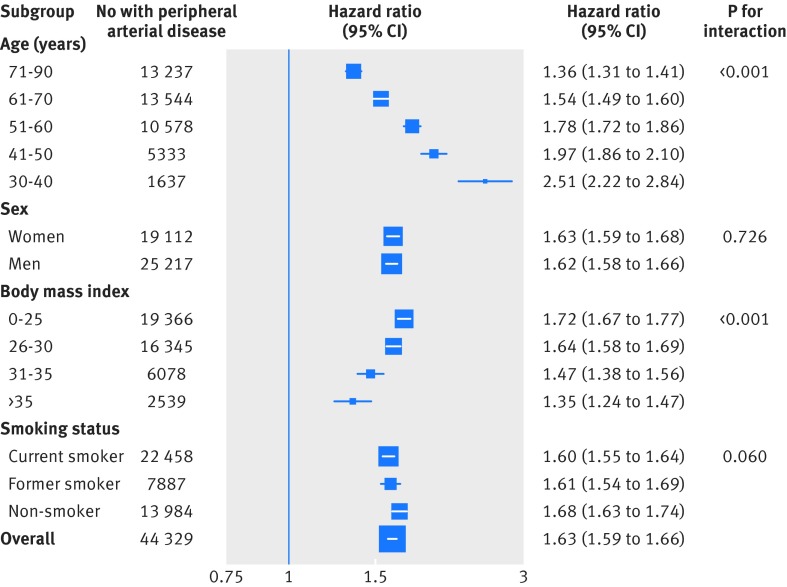
**Fig 2** Adjusted hazard ratios of 20 mm Hg higher usual systolic blood pressure for incident peripheral arterial disease stratified by patient subgroup. Adjustments were for age, body mass index, smoking status, sex, baseline diabetes, and baseline antihypertensive and lipid lowering drug use. For subgroups of age, adjustment was also for the interaction between systolic blood pressure and age category. For subgroups of sex, adjustment was also for the interaction between sex and systolic blood pressure. For subgroups of body mass index, adjustments were also for the interaction between systolic blood pressure and body mass index category. For subgroups of smoking, adjustments were also for the interaction between systolic blood pressure and smoking status

Six prospective studies that reported an association between blood pressure and risk of peripheral arterial disease were identified (see supplementary fig 10 and supplementary table 1). Although significant heterogeneity was observed between previous studies (I^2^=92.2%, see supplementary fig 1), a test for interaction between the pooled estimate of previous studies (relative risk 2.12, 95% confidence interval 1.20 to 3.76 for each 20 mm Hg higher systolic blood pressure) and the current estimate was insignificant (P=0.37). Overall, a 20 mm Hg higher systolic blood pressure was associated with a 92% higher risk of peripheral arterial disease (1.92, 1.38 to 2.65).

### Association of peripheral arterial disease with incident vascular disease

Baseline peripheral arterial disease was associated with a 51% higher risk of a vascular event (hazard ratio 1.51, 95% confidence interval 1.47 to 1.55, fig 3). However, substantial heterogeneity was observed by the subtype of vascular event. Risk of atrial fibrillation and chronic kidney disease was only mildly higher with peripheral arterial disease (1.25, 1.16 to 1.34 and relative risk 1.31, 95% confidence interval 1.25 to 1.38). In contrast, the risk of heart failure was increased by 63% (relative risk 1.63, 95% confidence interval 1.52 to 1.75) and ischaemic heart disease by 68% (1.68, 1.58 to 1.79). Although the risk of ischaemic stroke and unspecified stroke was increased with peripheral arterial disease, no association with haemorrhagic stroke was observed. The vascular event with the strongest association was aortic aneurysm, for which baseline peripheral arterial disease was associated with 2.1 times the risk (hazard ratio 2.10, 95% confidence interval 1.79 to 2.45). Estimates were similar in various sensitivity analyses (see supplementary figs 11-17 and supplementary table 6). Although a lower risk of haemorrhagic stroke was observed when the first four years of follow-up were excluded (see supplementary fig 13), this was not observed in any other sensitivity analyses, suggesting that this inverse association may be due to chance. The corresponding absolute increase in risk of any cardiovascular event associated with baseline peripheral arterial disease was 802 events per 100 000 person years of follow-up, respectively.

**Figure fig3:**
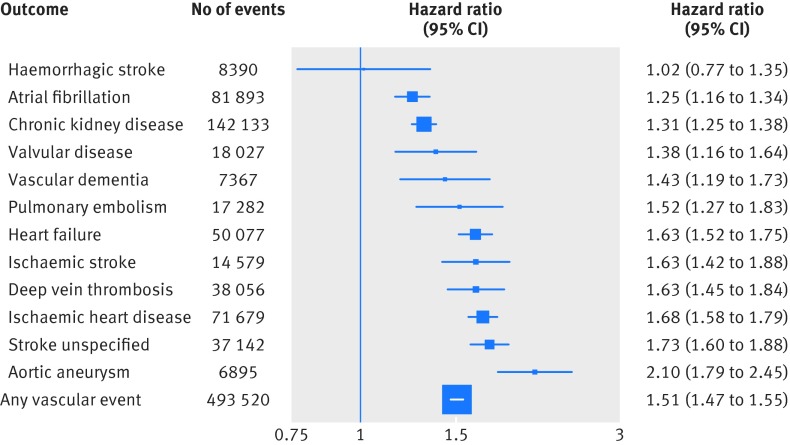
**Fig 3** Adjusted hazard ratios of baseline peripheral arterial disease versus no peripheral arterial disease for 12 different vascular events. Adjustments were for age, body mass index, smoking status, sex, baseline diabetes, baseline antihypertensive and lipid lowering drug use, and baseline peripheral arterial disease

The relative proportion of incident non-peripheral arterial disease vascular events was broadly similar among participants with and without peripheral arterial disease (table 2). Despite the modest association of incident chronic kidney disease with peripheral arterial disease, the most common initial vascular event among those with peripheral arterial disease was chronic kidney disease (24.4% of initial events) owing to the high incidence of chronic kidney disease in the non-peripheral arterial disease population (28.9% of initial events among those without peripheral arterial disease). Among participants with peripheral arterial disease, the second most common initial vascular event was ischaemic heart disease (18.5% of initial events), followed by heart failure (14.7%) and atrial fibrillation (13.2%). These four outcomes (chronic kidney disease, ischaemic heart disease, heart failure, and atrial fibrillation) comprised 70.8% of all initial vascular events among participants with peripheral arterial disease. The least common initial vascular event among participants with peripheral arterial disease was haemorrhagic stroke (1% of vascular events).

**Table 2 tbl2:** Distribution of first vascular event among participants with or without peripheral arterial disease at baseline. Values are numbers (percentages) of participants

Vascular events	Peripheral arterial disease at baseline	
	Present	Absent
Chronic kidney disease	1895 (24.4)	140 238 (28.9)
Ischaemic heart disease	1434 (18.5)	70 245 (14.5)
Heart failure	1143 (14.7)	48 934 (10.1)
Atrial fibrillation	1021 (13.2)	80 872 (16.6)
Unspecified stroke	838 (10.8)	36 304 (7.5)
Deep vein thrombosis	369 (4.8)	37 687 (7.8)
Aortic aneurysm	243 (3.1)	6652 (1.4)
Valvular heart disease	174 (2.2)	17 853 (3.7)
Ischaemic stroke	266 (3.4)	14313 (2.9)
Vascular dementia	155 (2.0)	7212 (1.5)
Pulmonary embolism	147 (1.9)	17 135 (3.5)
Haemorrhagic stroke	75 (1.0)	8315 (1.7)
All vascular events	7760 (100)	485 760 (100)

When observed risks of stroke (all subtypes), ischaemic heart disease, and vascular mortality were compared with previously reported relative risks from the Ankle Brachial Index Collaboration, no significant interaction was observed for stroke or vascular mortality (P=0.39 and P=0.73 for interaction, respectively, see supplementary fig 18). The risk of ischaemic heart disease was increased compared with the results of the Ankle Brachial Index Collaboration (relative risk 1.68, 95% confidence interval 1.58 to 1.79 *v*1.35, 1.10 to 1.65, P=0.04 for interaction). Overall, after random effects meta-analysis peripheral arterial disease was associated with a 64% higher risk of stroke (1.64, 1.54 to 1.75), a 54% higher risk of ischaemic heart disease (1.54, 1.25 to 1.90), and an 86% higher risk of vascular death (1.86, 1.75 to 1.99).

## Discussion

In this analysis of 4.2 million people and 44 329 incident peripheral arterial disease events, systolic blood pressure was observed to continuously relate to risk of peripheral arterial disease, with no evidence of a nadir in the range of 110 to 170 mm Hg. A 20 mm Hg higher systolic blood pressure was associated with a 63% increase in risk of peripheral arterial disease, whereas a 10 mm Hg higher diastolic blood pressure was associated with a 35% increase. Although the proportional association between systolic blood pressure and peripheral arterial disease declined with increasing age and body mass index, the association was not modified by sex or smoking status. Baseline peripheral arterial disease was observed to be a further risk factor for 11 different vascular events.

### Strengths and limitations of this study

This analysis has several strengths. The large size of this analysis (more than four million participants, more than 400 000 non-peripheral arterial disease vascular events, and more than 40 000 peripheral arterial disease events) allowed for the examination of associations in patient subpopulations (for example, by age and body mass index) and the examination of less common vascular events (for example, haemorrhagic stroke, pulmonary embolism). The population under study is also contemporary, with widespread use of antihypertensive and lipid lowering drugs.

This analysis also has several limitations. Firstly, we used information from electronic health records. Although the Clinical Practice Research Datalink database has been validated and used extensively for epidemiological research, including the relations between blood pressure, diabetes, and vascular disease,^11^ ^18^ the risk of misclassification of events may be greater than in a traditional cohort study. However, our estimates were consistent in several sensitivity analyses. Furthermore, we supplemented our analysis of Clinical Practice Research Datalink with a meta-analysis of prospective studies examining the association between blood pressure and peripheral arterial disease, and for analysis of the association between peripheral arterial disease and vascular disease we compared our estimates with a previous meta-analysis. This complementary approach allowed us to validate our overall estimates, while providing us with the power to examine associations in patient subpopulations and for less common vascular events. Secondly, people with hypertension and peripheral arterial disease may be more likely to be screened for cardiovascular disease than people without those disorders. However, analysis of cause specific mortality events (which are unlikely to be influenced by screening) resulted in similar estimates.

### Comparison with previous studies

Previous analyses of the relation between blood pressure and peripheral arterial disease have largely been limited to cross sectional studies.^1^ ^7^ Of the six previous prospective cohort studies that were identified, five observed a significant positive association between blood pressure and peripheral arterial disease, whereas one observed a negative association (see supplementary fig 1). Our results of a positive association between blood pressure and peripheral arterial disease are consistent with our meta-analysis of these previous studies, and our observation of an interaction of the association between blood pressure and risk of peripheral arterial disease by age is consistent with a previous analysis of data from the Clinical Practice Research Datalink.^11^ Our demonstration of an interaction effect by body mass index but not by sex or smoking status, extends these previous studies. In particular, the lack of an interaction observed by smoking status highlights the importance of adequate blood pressure control for prevention of peripheral arterial disease in both smokers and non-smokers. Given that current smoking is associated with 4.5 times the risk of prevalent peripheral arterial disease,^7^ the absolute difference in risk associated with a 20 mm Hg higher systolic blood pressure in current smokers will be approximately 4.5 times the associated risk in non-smokers. Similarly, while proportional associations declined with increasing age and body mass index, the absolute risk difference increased, owing to the much greater absolute risk of peripheral arterial disease with increasing age and body mass index.

In the second analysis of this study, peripheral arterial disease was observed to be a risk factor for 11 different vascular events. However, the strength of the association between baseline peripheral arterial disease and initial vascular disease varied significantly, from a 25% higher risk of atrial fibrillation to a 110% higher risk of aortic aneurysm. Baseline peripheral arterial disease was also associated with a significantly higher risk of ischaemic heart disease and unspecified stroke. Despite the modestly higher risk of chronic kidney disease associated with peripheral arterial disease (hazard ratio 1.31, 95% confidence interval 1.25 to 1.38), chronic kidney disease was the most common initial vascular event among people with peripheral arterial disease, owing to the high absolute risk of the event in populations with non-peripheral arterial disease. Overall, 71% of initial vascular events were one of chronic kidney disease, ischaemic heart disease, heart failure, and atrial fibrillation.

Previous analyses of the association between peripheral arterial disease and other vascular events have largely focused on peripheral arterial disease and ischaemic heart disease or stroke. In the Ankle Brachial Index Collaboration, peripheral arterial disease was associated with a 64% higher risk of stroke (relative risk 1.64, 95% confidence interval 1.54 to 1.75), a 54% higher risk of ischaemic heart disease (1.54, 1.25 to 1.90), and an 86% higher risk of vascular death (1.86, 1.75 to 1.99).^12^ Our results were broadly similar to those of this meta-analysis of traditional cohort studies, with no significant interaction between our estimate and the previous meta-analysis’s estimate observed for stroke or vascular mortality and a mildly increased risk of ischaemic heart disease observed in our population compared with the Ankle Brachial Index Collaboration’s estimate.^12^ Our demonstration of associations between peripheral arterial disease and several vascular events, including aortic aneurysm, ischaemic stroke, chronic kidney disease, valvular heart disease, heart failure, vascular dementia, deep vein thrombosis, and venous thromboembolism, extends this and previous analyses^19^ ^20^ ^21^ and provides reliable quantification of the strength of the association of peripheral arterial disease with less commonly reported vascular events.

### Clinical and research implications

Our results have several clinical and research implications. The strong continuous association of baseline blood pressure with incident peripheral arterial disease suggests that blood pressure lowering may reduce the risk of peripheral arterial disease. An individual patient data meta-analysis, such as the Blood Pressure Lowering Treatment Trialists’ Collaboration, could confirm this observation and examine whether effects differ in important patient subpopulations, such as current smokers. The reliable estimation of the association of both systolic and diastolic blood pressure with incident peripheral arterial disease, adjusted for regression dilution bias, may also allow for the improved design of clinical trials aiming to examine the effects of blood pressure lowering on peripheral arterial disease. The subgroup specific associations of blood pressure with peripheral arterial disease may inform communication of risks associated with raised blood pressure with patients.

Our results also highlight the importance of tailoring treatment in peripheral arterial disease beyond the prevention of stroke and ischaemic heart disease. Indeed, both represented only one third (33.7%) of initial presentations of vascular disease among those with peripheral arterial disease. As the treatments most effective for the prevention of atrial fibrillation, chronic kidney disease, or heart failure may differ from those most effective for the prevention of ischaemic heart disease and stroke (for example, calcium channel blockers are less effective at preventing heart failure than are other blood pressure lowering drugs),^22^ these results suggest that guidelines focused on the management of peripheral arterial disease should consider the heightened risk that people with peripheral arterial disease have for a variety of vascular events, beyond stroke and ischaemic heart disease.^23^ Recent large trials in peripheral arterial disease have largely used ischaemic heart disease and stroke as outcomes, while excluding other vascular events, including heart failure, chronic kidney disease, or atrial fibrillation, as adjudicated endpoints.^24^ ^25^ Our results suggest that trials conducted in populations with peripheral arterial disease should consider including other vascular events as outcomes, particularly heart failure, atrial fibrillation, and chronic kidney disease (which in combination represent more than half of initial vascular events among those with peripheral arterial disease).

### Conclusions

A 20 mm Hg higher than usual systolic blood pressure is associated with a 63% higher risk of peripheral arterial disease. This association is modified by age and body mass index but not by sex and smoking status. Peripheral arterial disease is a further risk factor for atrial fibrillation, chronic kidney disease, valvular heart disease, vascular dementia, pulmonary embolism, heart failure, ischaemic stroke, deep vein thrombosis, ischaemic heart disease, unspecified stroke, and aortic aneurysm. However, the most common initial presentation of vascular disease among those with peripheral arterial disease was chronic kidney disease, followed by ischaemic heart disease, heart failure, and atrial fibrillation.

**What is already known on this topic**

Limited evidence suggests that raised blood pressure is associated with an increased risk of peripheral arterial disease and that the disease is associated with an increased risk of ischaemic heart disease and stroke
However, previous analyses of the association of blood pressure with risk of peripheral arterial disease have not been sufficiently large to reliably investigate the associations in more detail

**What this study adds**

In this large scale analysis of a contemporary cohort, each 20 mm Hg higher systolic blood pressure was associated with a 63% higher risk of peripheral arterial disease
The strength of the association declined with increasing age and body mass index but was not modified by sex or smoking status
Peripheral arterial disease was further associated with a wide range of incident vascular diseases, including ischaemic heart disease, heart failure, aortic aneurysm, chronic kidney disease, and stroke

## Notes

Ethical approval: The CPRD Group has obtained ethical approval from a National Research Ethics Service Committee for all purely observational research using anonymised Clinical Practice Research Datalink data. Separate ethical approval for this study was not required.

Data sharing: The code is available from the lead author on request. Access to Clinical Practice Research Datalink data requires application to the Independent Scientific Advisory Committee (www.cprd.com).

Transparency: The manuscripts guarantors (CAE and KR) affirm that the manuscript is an honest, accurate, and transparent account of the study being reported; that no important aspects of the study have been omitted; and that any discrepancies from the study as planned (and, if relevant, registered) have been explained.

## Notes

Cite this as: *BMJ* 2015;351:h4865

## References

[1] Fowkes FGR, Rudan D, Rudan I, et al. Comparison of global estimates of prevalence and risk factors for peripheral artery disease in 2000 and 2010: a systematic review and analysis. Lancet 2013;382:1329-40. 23915883 10.1016/S0140-6736(13)61249-0

[2] Gregg EW, Sorlie P, Paulose-Ram R, et al. Prevalence of lower-extremity disease in the US adult population >=40 years of age with and without diabetes: 1999-2000 national health and nutrition examination survey. Diabetes Care 2004;27:1591-7. 15220233 10.2337/diacare.27.7.1591

[3] Ankle Brachial Index Collaboration, Fowkes FGR, Murray GD, et al. Ankle brachial index combined with Framingham Risk Score to predict cardiovascular events and mortality: a meta-analysis. JAMA 2008;300:197-208. 18612117 10.1001/jama.300.2.197 PMC2932628

[4] Gu Q, Burt VL, Dillon CF, et al. Trends in antihypertensive medication use and blood pressure control among United States adults with hypertension: the National Health And Nutrition Examination Survey, 2001 to 2010. Circulation 2012;126:2105-14. 23091084 10.1161/CIRCULATIONAHA.112.096156

[5] Walley T, Folino-Gallo P, Stephens P, et al. Trends in prescribing and utilization of statins and other lipid lowering drugs across Europe 1997-2003. Br J Clin Pharmacol 2005;60:543-51. 16236045 10.1111/j.1365-2125.2005.02478.x PMC1884951

[6] Murray CJL, Vos T, Lozano R, et al. Disability-adjusted life years (DALYs) for 291 diseases and injuries in 21 regions, 1990-2010: a systematic analysis for the Global Burden of Disease Study 2010. Lancet 2012;380:2197-223. 23245608 10.1016/S0140-6736(12)61689-4

[7] Selvin E, Erlinger TP. Prevalence of and risk factors for peripheral arterial disease in the United States: results from the National Health and Nutrition Examination Survey, 1999-2000. Circulation 2004;110:738-43. 15262830 10.1161/01.CIR.0000137913.26087.F0

[8] Rahimi K, Emdin C, MacMahon S. The epidemiology of blood pressure and its worldwide management. Circ Res 2015;116:925-36. 25767281 10.1161/CIRCRESAHA.116.304723

[9] Adler AI, Stevens RJ, Neil A, et al. UKPDS 59: hyperglycemia and other potentially modifiable risk factors for peripheral vascular disease in type 2 diabetes. Diabetes Care 2002;25:894-9. 11978687 10.2337/diacare.25.5.894

[10] Cockcroft JR, Wilkinson IB, Evans M, et al. Pulse pressure predicts cardiovascular risk in patients with type 2 diabetes mellitus. Am J Hypertens 2005;18:1463-7-discussion1468-9. 16280282 10.1016/j.amjhyper.2005.05.009

[11] Rapsomaniki E, Timmis A, George J, et al. Blood pressure and incidence of twelve cardiovascular diseases: lifetime risks, healthy life-years lost, and age-specific associations in 1·25 million people. Lancet 2014;383:1899-911. 24881994 10.1016/S0140-6736(14)60685-1 PMC4042017

[12] Heald CL, Fowkes FGR, Murray GD, et al. Risk of mortality and cardiovascular disease associated with the ankle-brachial index: systematic review. Atherosclerosis 2006;189:61-9. 16620828 10.1016/j.atherosclerosis.2006.03.011

[13] Herrett E, Shah AD, Boggon R, et al. Completeness and diagnostic validity of recording acute myocardial infarction events in primary care, hospital care, disease registry, and national mortality records: cohort study. BMJ 2013;346:f2350-0. 23692896 10.1136/bmj.f2350 PMC3898411

[14] Springate DA, Kontopantelis E, Ashcroft DM, et al. ClinicalCodes: an online clinical codes repository to improve the validity and reproducibility of research using electronic medical records. PLoS One 2014;9:e99825. 24941260 10.1371/journal.pone.0099825 PMC4062485

[15] Clarke R, Shipley M, Lewington S, et al. Underestimation of risk associations due to regression dilution in long-term follow-up of prospective studies. Am J Epidemiol 1999;150:341-53. 10453810 10.1093/oxfordjournals.aje.a010013

[16] Wormser D, White IR, Thompson SG, et al. Within-person variability in calculated risk factors: comparing the aetiological association of adiposity ratios with risk of coronary heart disease. Int J Epidemiol 2013;42:849-59. 23918853 10.1093/ije/dyt077 PMC3733701

[17] Baker JF, Schumacher HR, Krishnan E. Serum uric acid level and risk for peripheral arterial disease: analysis of data from the multiple risk factor intervention trial. Angiology 2007;58:450-7. 17875958 10.1177/0003319707303444

[18] Shah AD, Langenberg C, Rapsomaniki E, et al. Type 2 diabetes and incidence of cardiovascular diseases: a cohort study in 1·9 million people. Lancet Diabetes Endocrinol 2015;3:105-13. 25466521 10.1016/S2213-8587(14)70219-0 PMC4303913

[19] Leng GC, Fowkes FG, Lee AJ, et al. Use of ankle brachial pressure index to predict cardiovascular events and death: a cohort study. BMJ 1996;313:1440-4. 8973232 10.1136/bmj.313.7070.1440 PMC2352992

[20] Golomb BA, Dang TT, Criqui MH. Peripheral arterial disease: morbidity and mortality implications. Circulation 2006;114:688-99. 16908785 10.1161/CIRCULATIONAHA.105.593442

[21] Diehm C, Lange S, Darius H, et al. Association of low ankle brachial index with high mortality in primary care. Eur Heart J 2006;27:1743-9. 16782720 10.1093/eurheartj/ehl092

[22] Law MR, Morris JK, Wald NJ. Use of blood pressure lowering drugs in the prevention of cardiovascular disease: meta-analysis of 147 randomised trials in the context of expectations from prospective epidemiological studies. BMJ 2009;338:b1665. 19454737 10.1136/bmj.b1665 PMC2684577

[23] 2011 Writing Group Members, 2005 Writing Committee Members, ACCF/AHA Task Force Members. 2011 ACCF/AHA Focused Update of the Guideline for the Management of patients with peripheral artery disease (Updating the 2005 Guideline): a report of the American College of Cardiology Foundation/American Heart Association Task Force on practice guidelines. Circulation 2011;124:2020-45. 21959305 10.1161/CIR.0b013e31822e80c3

[24] Belch J, MacCuish A, Campbell I, et al. The prevention of progression of arterial disease and diabetes (POPADAD) trial: factorial randomised placebo controlled trial of aspirin and antioxidants in patients with diabetes and asymptomatic peripheral arterial disease. BMJ 2008;337:a1840-0. 18927173 10.1136/bmj.a1840 PMC2658865

[25] Warfarin Antiplatelet Vascular Evaluation Trial Investigators, Anand S, Yusuf S, et al. Oral anticoagulant and antiplatelet therapy and peripheral arterial disease. N Engl J Med 2007;357:217-27. 17634457 10.1056/NEJMoa065959

