# Supplementary material for: Usual blood pressure, peripheral arterial disease, and vascular risk: cohort study of 4.2 million adults
Source: BMJ. 2015 Sep 29;351:h4865. doi: 10.1136/bmj.h4865 (PMC4586462; doi:10.1136/bmj.h4865)
Supplement: Supplementary file 1 [file emdc027545.ww1_default.pdf]

Supplementary Appendix

Supp. Table 1. Characteristics of prospective studies included in meta-analysis that associated blood pressure with incident peripheral arterial disease. Only studies that could be standardized per 20 mm Hg higher systolic blood pressure were included. Note that Rapsomaniki et al.[1] was excluded from the meta-analysis due to an overlapping cohort with the current study.

Study (Author, Year)	Name of Cohort(s)	Location	Baseline Year	Population	Participants	Incident PAD	Adjustments	Median Follow Up (Years)
Previous cohort studies								
Adler, 2002[2]	UKPDS	United Kingdom	1977-1991	Adults aged 25– 65 years with newly diagnosed type 2 diabetes	2398	61	Age, SBP, HbA1c, other clinical variables	8.9
Baena-Diez, 2007[3]	Barcelona	Barcelona, Spain	1973	General population without cardiovascular events	932	17	Age, sex, smoking, total and HDL cholesterol, and diabetes mellitus	8
Baker, 2007[4]	MRFIT	United States	1996	Men aged 35 to 57, at high risk of CAD	12828	283	Age, cholesterol, BMI, serum uric acid, other clinical variables	10.5
Cockcroft, 2005[5]	Cardiff Diabetes Database	Cardiff, United Kingdom	1992-1995	Adults with diabetes	2911	157	Age, sex, smoking, total cholesterol/HDL cholesterol	4
Kannel, 1972[6]	Framingham	Framingham, United States	1973	Adults aged 45-74	5209	122	Cholesterol, glucose intolerance, age, cigarettes	16
Powell, 2011[7]	Women's Health Study	United States	1949	Women aged ≥ 45 years without prior cancer or CVD	39260	178	Age, BMI, hyperlipidemia, diabetes, other clinical/demographic characteristics	13.3
Current study								
Emdin, 2015	CPRD	United Kingdom	1990-2013	Adults aged 30 to 90, free of cardiovascular disease	4204190	44329	Age, sex, BMI, diabetes, antihypertensive use, statins	7.0

Supp. Table 2. Primary and secondary endpoints used in the analysis.

	Definition	ICD-10 Codes	ICD-9 Codes	Prior Validation Studies of Disease in CPRD
Primary outcome				
Peripheral arterial disease	Incident diagnosis of peripheral arterial disease, including intermittent claudication, hospitalization or death due to peripheral arterial disease.	I73.1, I73.8, I73.9, I74.3, I74.4, I74.5 (fatal and non-fatal)	443.1, 443.8, 443.9, 444.22, 444.81 (fatal and non-fatal)	Similar association with cardiovascular risk factors as traditional cohort studies.[1,8] Median PPV of 85% for GPRD coding of circulatory disorders.[9]
Secondary outcomes				
Atrial fibrillation	Incident diagnosis of, hospitalization or death due to atrial fibrillation or atrial flutter, no restrictions on type.	I48 (fatal and non-fatal)	427.3 (fatal and non-fatal),	Similar association with risk factors as from traditional cohort studies.[10]
Aortic aneurysm	Incident diagnosis of, hospitalization or death due to aortic aneurysm.	I71 (fatal and non-fatal)	441 (fatal and non-fatal),	NA
Chronic kidney disease	Incident diagnosis of, hospitalization or death due to stage 3-5 chronic kidney disease.	N18, I12, I13, E08.22, E09.22, E10.22, E11.22, E13.22 (fatal and non-fatal)	403, 404 (fatal and non-fatal)	Similar prevalence in CPRD to national cross-sectional surveys.[11]
Diabetes	Incident diagnosis of, hospitalization or death due to diabetes or prescription of antidiabetic medication.	E11 (fatal and non-fatal)	250 (fatal and non-fatal)	PPV: 98.6%[12]
Deep vein thrombosis	Incident diagnosis of, hospitalization or death due to deep vein thrombosis.	I80-I82 (fatal and non-fatal)	451-453 (fatal and non-fatal)	PPV for VTE (combined DVT and PE): 88.2%[12]
Pulmonary embolism	Incident diagnosis of, hospitalization or death due to pulmonary embolism.	I26 (fatal and non-fatal)	415.1	
Ischemic heart disease	Incident diagnosis of, hospitalization non-fatal myocardial infarction or death due to ischemic heart disease.	I20-I25(fatal), I21-I23(non-fatal)	410-414(fatal), 410-412 (fatal and non-fatal)	PPV: 92.6%[12]
Heart failure	Incident diagnosis of, hospitalization or death due to heart failure.	I11.0, I13.0, I13.2, I50 (fatal and non-fatal)	428, 402.01, 402.11, 402.91, 404.01, 404.11, 404.91, 404.03, 404.13 404.93 (fatal and non-fatal)	PPV: 81.8%[13]
Stroke	Incident diagnosis of,	I60, I61, I63,	430, 431, 434,	PPV: 92.7%[12]

	hospitalization or death due to stroke.	I64 (fatal and non-fatal), I67.2, I67.9 (fatal only)	437 (fatal and non-fatal)	
Valvular Heart Disease	Incident diagnosis of, hospitalization or death due to valvular heart disease.	I34-I37 (fatal and non-fatal)	424 (fatal and non-fatal)	NA
Vascular dementia	Incident diagnosis of, hospitalization or death due to vascular dementia, including concomitant diagnoses of Alzheimer's disease and vascular dementia (i.e. mixed dementias).	F01 (fatal and non-fatal)	290.4 (fatal and non-fatal)	PPV: 75%[14]

Supp. Table 3. Proportion of different ICD codes at hospitalization for peripheral arterial disease at different baseline age groups.

Age	I73.1	I73.8	I73.9	I74.3	I74.4	I74.5
(29,40]	1.2%	3.2%	76.4%	11.5%	3.5%	4.1%
(40,50]	0.7%	1.8%	80.8%	11.4%	1.7%	3.5%
(50,60]	0.1%	1.0%	85.6%	10.6%	0.4%	2.4%
(60,70]	0.1%	0.7%	84.9%	12.2%	0.6%	1.4%
(70,90]	0.2%	1.0%	85.6%	11.4%	1.0%	0.8%

Supp. Table 4. Association of different units of blood pressure with risk of incident PAD.

SBP	HR (CI)	DBP	HR (CI)
20 mm Hg	1.63 (1.59, 1.66)	10 mm Hg	1.35 (1.31, 1.38)
10 mm Hg	1.28 (1.26, 1.29)	5 mm Hg	1.16 (1.14, 1.17)
5 mm Hg	1.13 (1.12, 1.14)	2.5 mm Hg	1.08 (1.07, 1.08)
2.5 mm Hg	1.06 (1.06, 1.07)	1.25 mm Hg	1.04 (1.03, 1.04)
Standard deviation (23.34 mm Hg)	1.77 (1.72, 1.81)	Standard deviation (11.57 mm Hg)	1.42 (1.37, 1.45)

Supp. Table 5. Association of usual systolic blood pressure with incident peripheral arterial disease in six sensitivity analyses (described in Supp. Figures 3-9).

**Sensitivity analyses**	**HR (CI)**
No sensitivity analysis	1.63 (1.59, 1.66)
1. Adjustment for cholesterol	1.61 (1.58, 1.65)
2. Adjustment for cholesterol and period of blood pressure measurement	1.60 (1.57, 1.63)
3. Exclusion of first two years of follow up	1.62 (1.58, 1.65)
4. Exclusion of first four years of follow up	1.58 (1.54, 1.63)
5. Exclusion of individuals prescribed blood pressure lowering drugs at baseline	1.68 (1.64, 1.72)
6. Cause-specific mortality events only	1.49 (1.23, 1.80)

Supp. Table 6. Association of incident cardiovascular disease with baseline peripheral arterial disease in seven sensitivity analyses (described in Supp. Figures 10-17).

**Sensitivity analyses**	**HR (CI)**
No sensitivity analysis	1.51 (1.47, 1.55)
1. Adjustment for cholesterol	1.49 (1.46, 1.53)
2. Adjustment for cholesterol and period of blood pressure measurement	1.50 (1.46, 1.54)
3. Exclusion of first two years of follow up	1.50 (1.45, 1.55)
4. Exclusion of first four years of follow up	1.46 (1.40, 1.52)
5. Peripheral arterial disease diagnosed earlier than three years prior is excluded	1.52 (1.47, 1.58)
6. Individuals with diagnoses of peripheral arterial disease after baseline blood pressure measurement are excluded	1.55 (1.51, 1.59)
7. Cause-specific mortality events only	1.86 (1.74, 1.99)

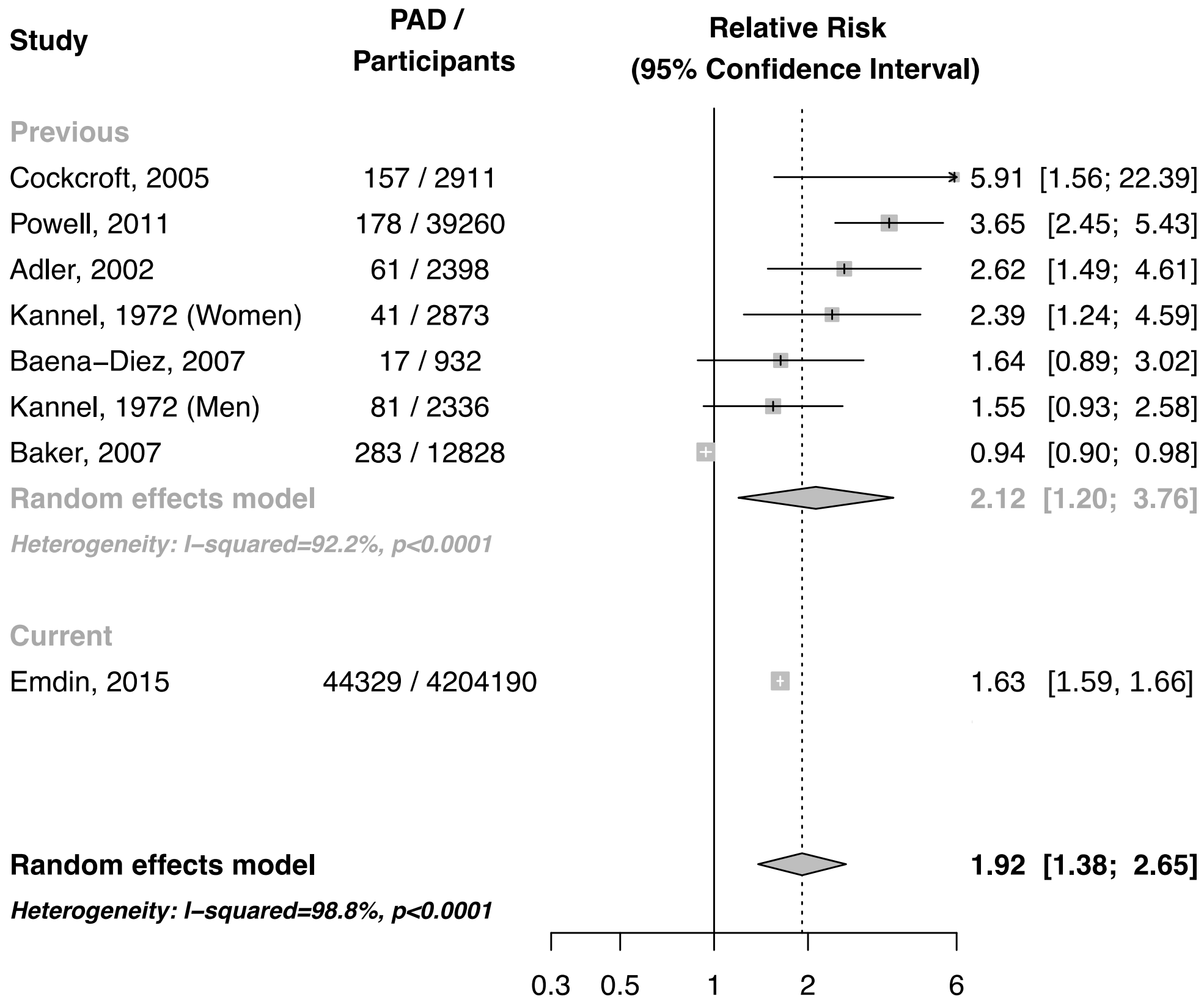

Supp. Figure 1. Risk of peripheral arterial disease per 20 mm Hg higher measured systolic blood pressure.

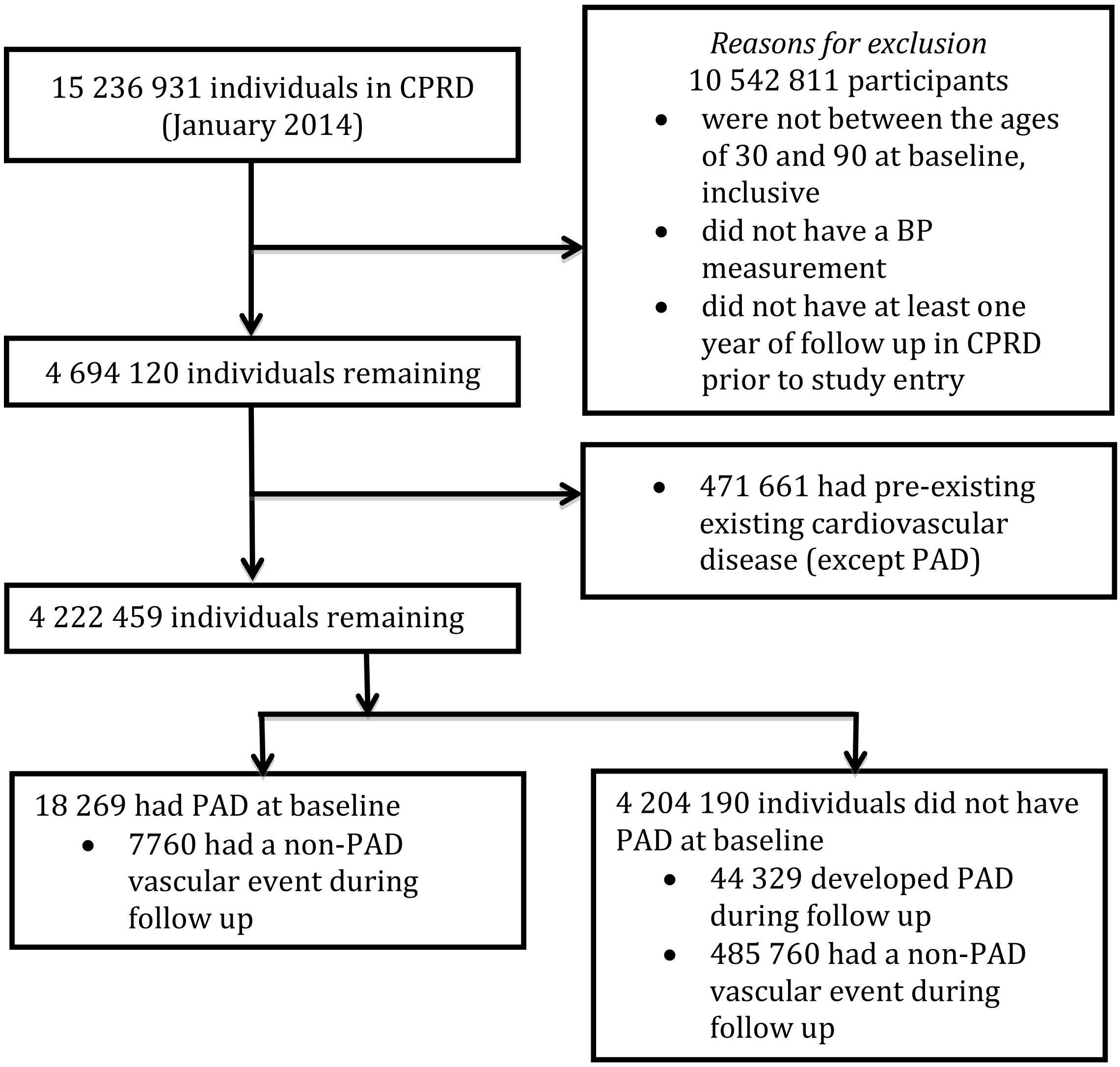

Supp. Figure 2. Flowchart of cohort identification.

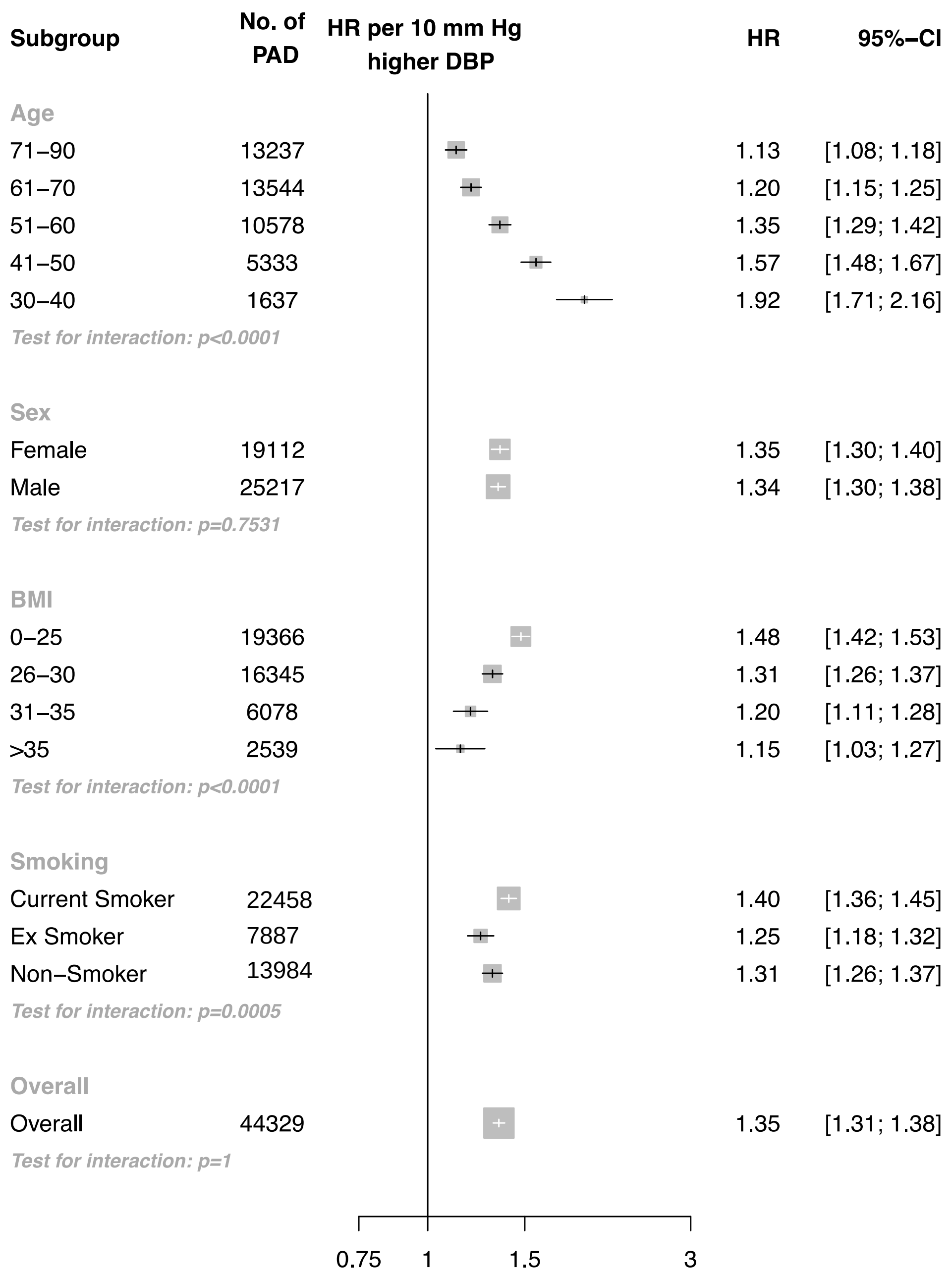

Supp. Figure 3. Adjusted hazard ratios of 10 mm Hg higher usual diastolic blood pressure for incident peripheral arterial disease stratified by patient subgroup. Adjustments were for age, body mass index, smoking status, sex, baseline diabetes, baseline antihypertensive use and baseline lipid lowering drug use. For subgroups of age, adjustment was also for age category and the interaction between systolic blood pressure and age category (hazard ratio shown). For subgroups of sex, adjustment was also for the interaction between sex and systolic blood pressure (hazard ratio shown). For subgroups of body mass index, adjustments were also for body mass index category and the interaction between systolic blood pressure and body mass index category (hazard ratio shown). Area of each square is proportional to the inverse variance of the estimate.

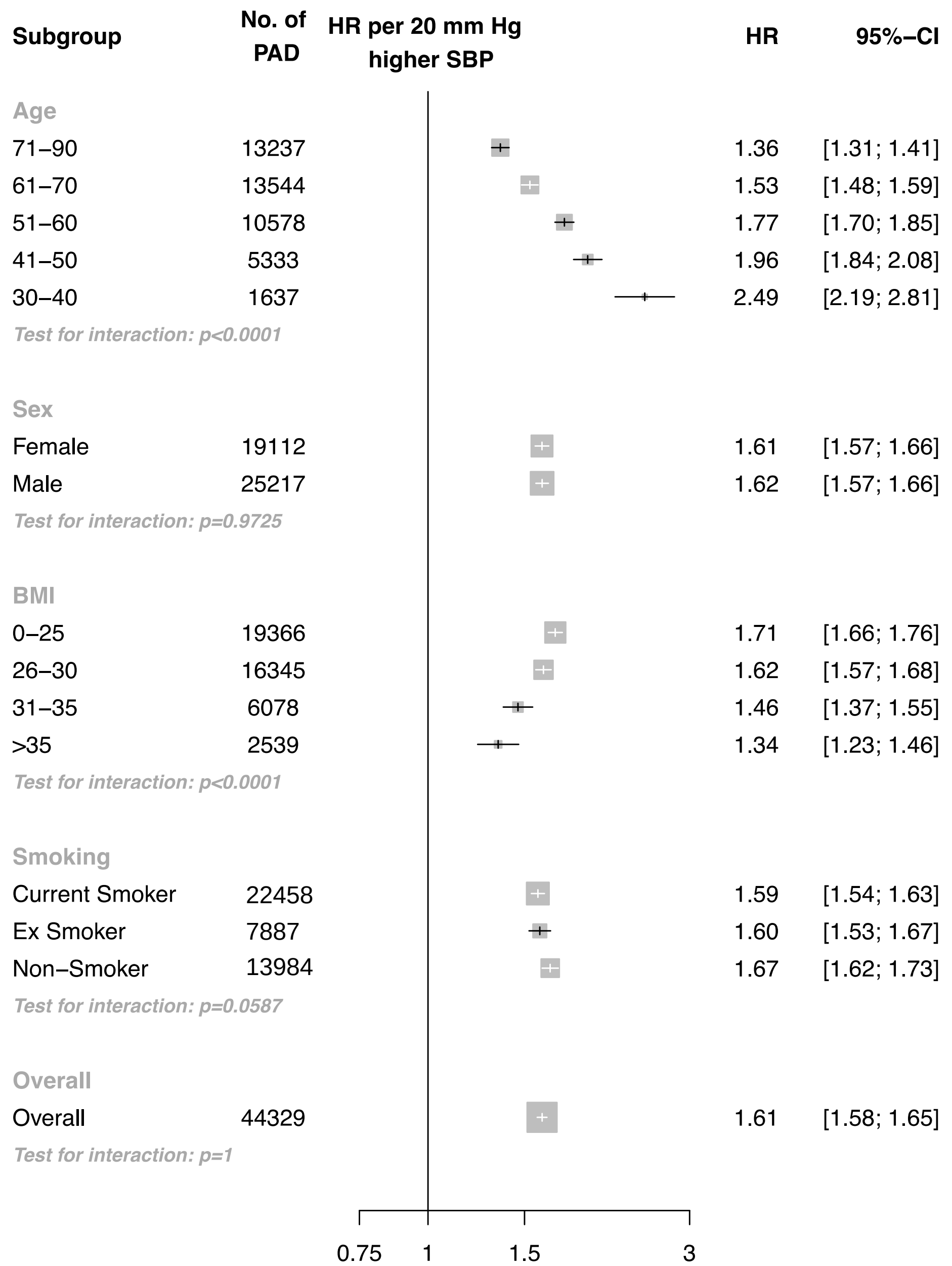

Supp. Figure 4. Adjusted hazard ratios of 20 mm Hg higher usual systolic blood pressure for incident peripheral arterial disease stratified by patient subgroup. Adjustments were for age, body mass index, smoking status, sex, baseline diabetes, baseline antihypertensive use, baseline lipid lowering drug use, total cholesterol and high density lipoprotein cholesterol. For subgroups of age, adjustment was also for age category and the interaction between systolic blood pressure and age category (hazard ratio shown). For subgroups of sex, adjustment was also for the interaction between sex and systolic blood pressure (hazard ratio shown). For subgroups of body mass index, adjustments were also for body mass index category and the interaction between systolic blood pressure and body mass index category (hazard ratio shown). Area of each square is proportional to the inverse variance of the estimate.

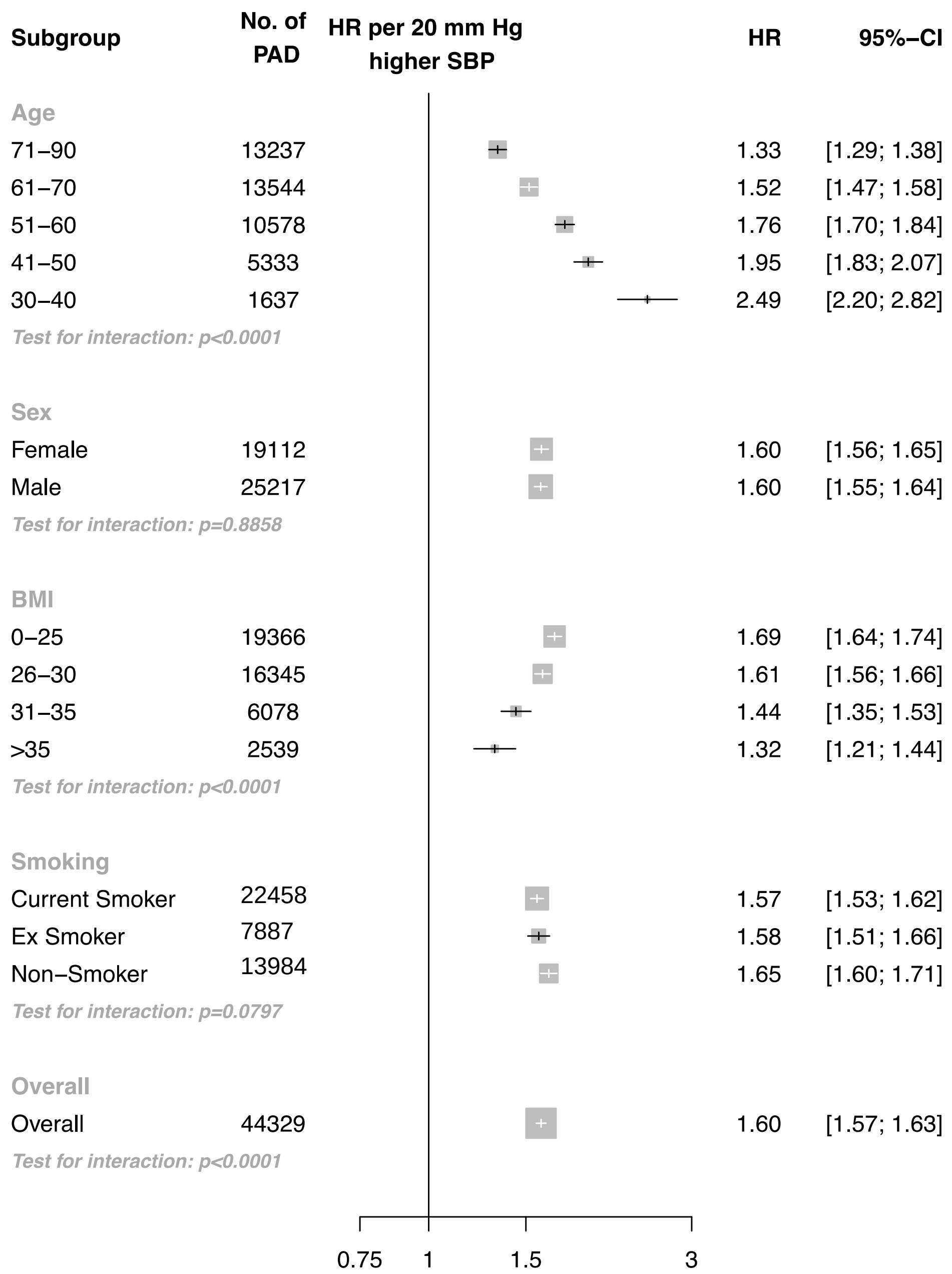

Supp. Figure 5. Adjusted hazard ratios of 20 mm Hg higher usual systolic blood pressure for incident peripheral arterial disease stratified by patient subgroup. Adjustments were for age, body mass index, smoking status, sex, baseline diabetes, baseline antihypertensive use, baseline lipid lowering drug use, total cholesterol, high density lipoprotein cholesterol and period of blood pressure measurement. For subgroups of age, adjustment was also for age category and the interaction between systolic blood pressure and age category (hazard ratio shown). For subgroups of sex, adjustment was also for the interaction between sex and systolic blood pressure (hazard ratio shown). For subgroups of body mass index, adjustments were also for body mass index category and the interaction between systolic blood pressure and body mass index category (hazard ratio shown). Area of each square is proportional to the inverse variance of the estimate.

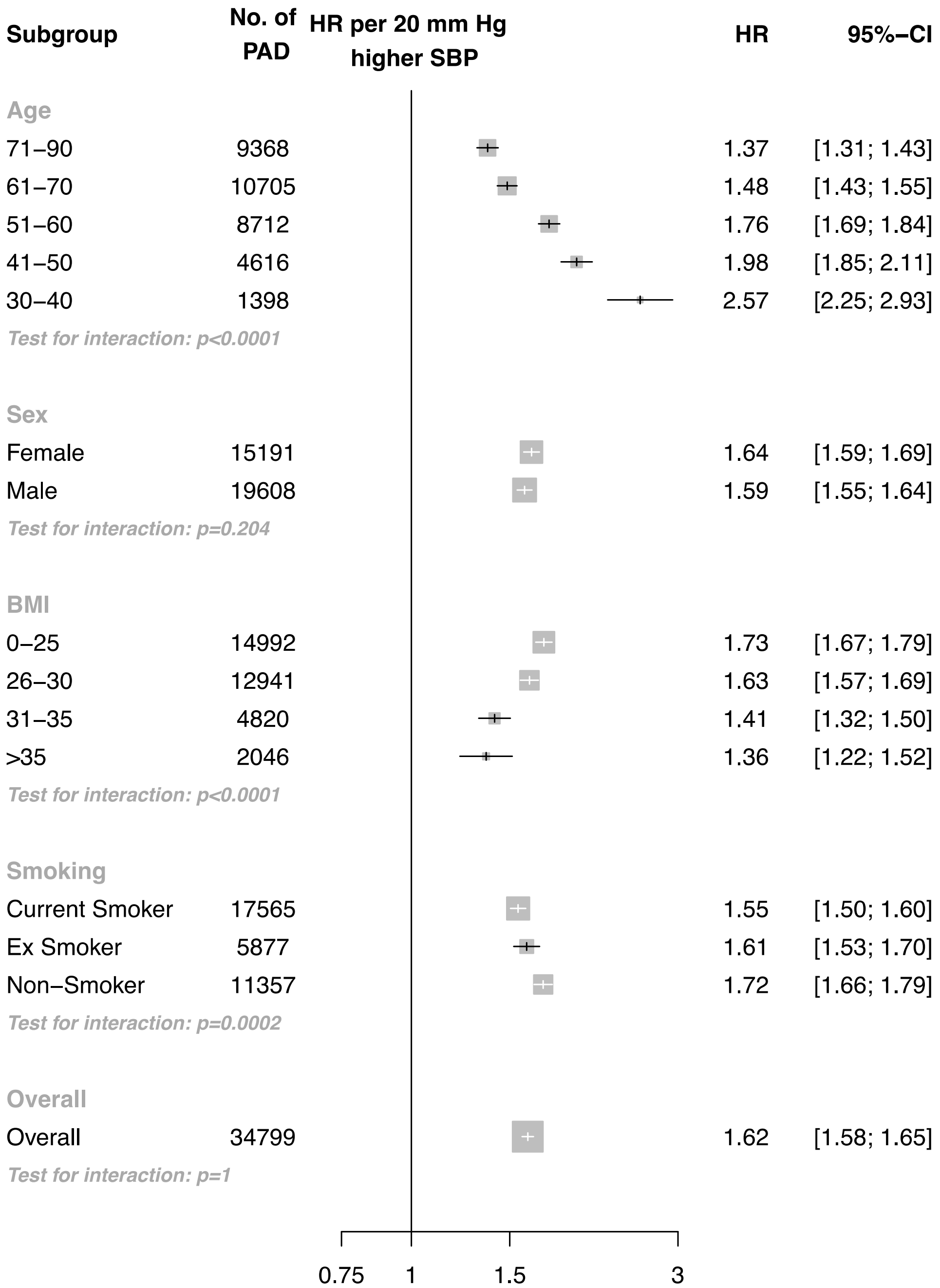

Supp. Figure 6. Adjusted hazard ratios of 20 mm Hg higher usual systolic blood pressure for incident peripheral arterial disease stratified by patient subgroup. Adjustments were for age, body mass index, smoking status, sex, baseline diabetes, baseline antihypertensive use and baseline lipid lowering drug use. For subgroups of age, adjustment was also for age category and the interaction between systolic blood pressure and age category (hazard ratio shown). For subgroups of sex, adjustment was also for the interaction between sex and systolic blood pressure (hazard ratio shown). For subgroups of body mass index, adjustments were also for body mass index category and the interaction between systolic blood pressure and body mass index category (hazard ratio shown). Area of each square is proportional to the inverse variance of the estimate. The first two years of follow up were excluded.

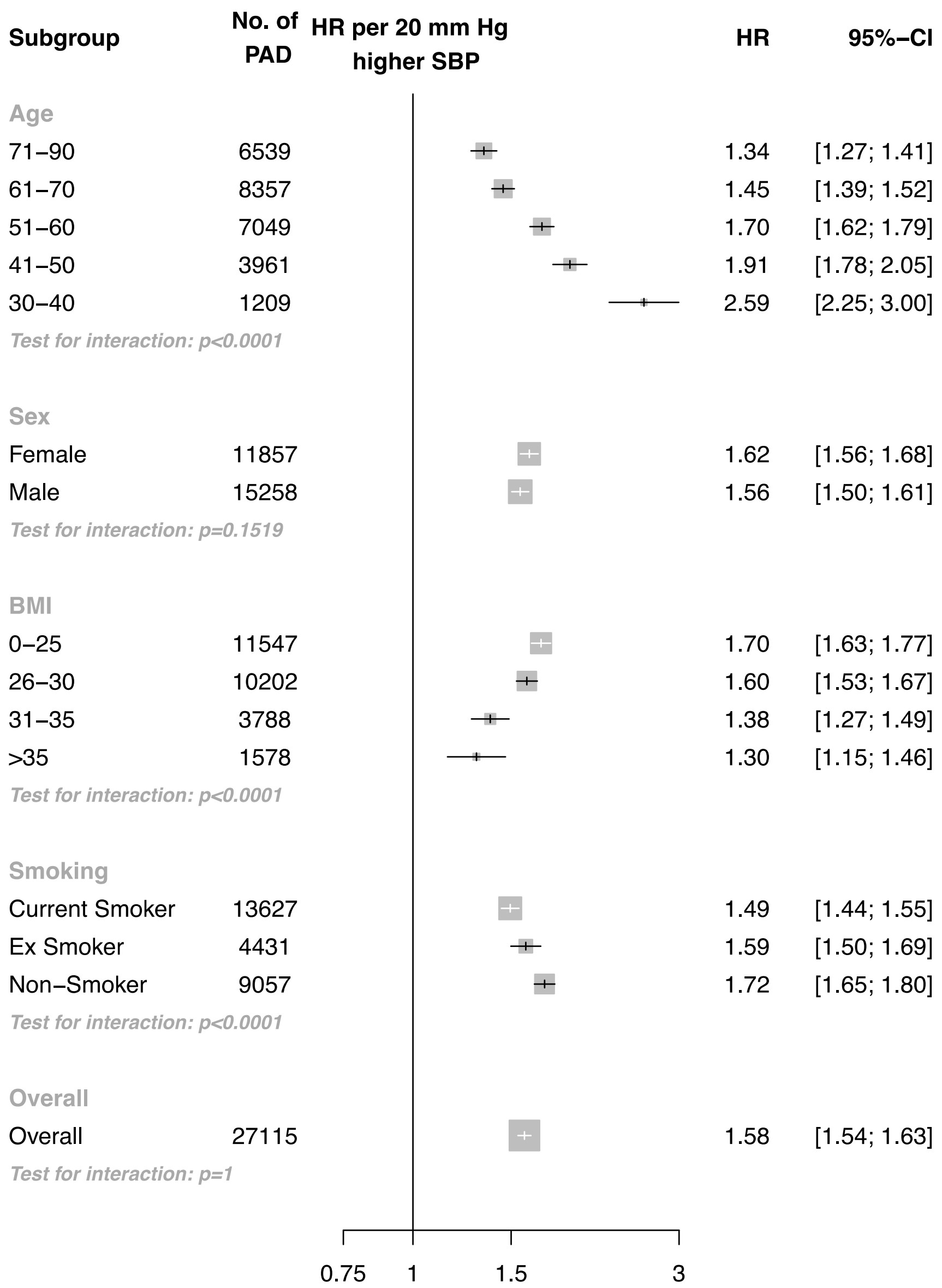

Supp. Figure 7. Adjusted hazard ratios of 20 mm Hg higher usual systolic blood pressure for incident peripheral arterial disease stratified by patient subgroup. Adjustments were for age, body mass index, smoking status, sex, baseline diabetes, baseline antihypertensive use and baseline lipid lowering drug use. For subgroups of age, adjustment was also for age category and the interaction between systolic blood pressure and age category (hazard ratio shown). For subgroups of sex, adjustment was also for the interaction between sex and systolic blood pressure (hazard ratio shown). For subgroups of body mass index, adjustments were also for body mass index category and the interaction between systolic blood pressure and body mass index category (hazard ratio shown). Area of each square is proportional to the inverse variance of the estimate. The first four years of follow up were excluded.

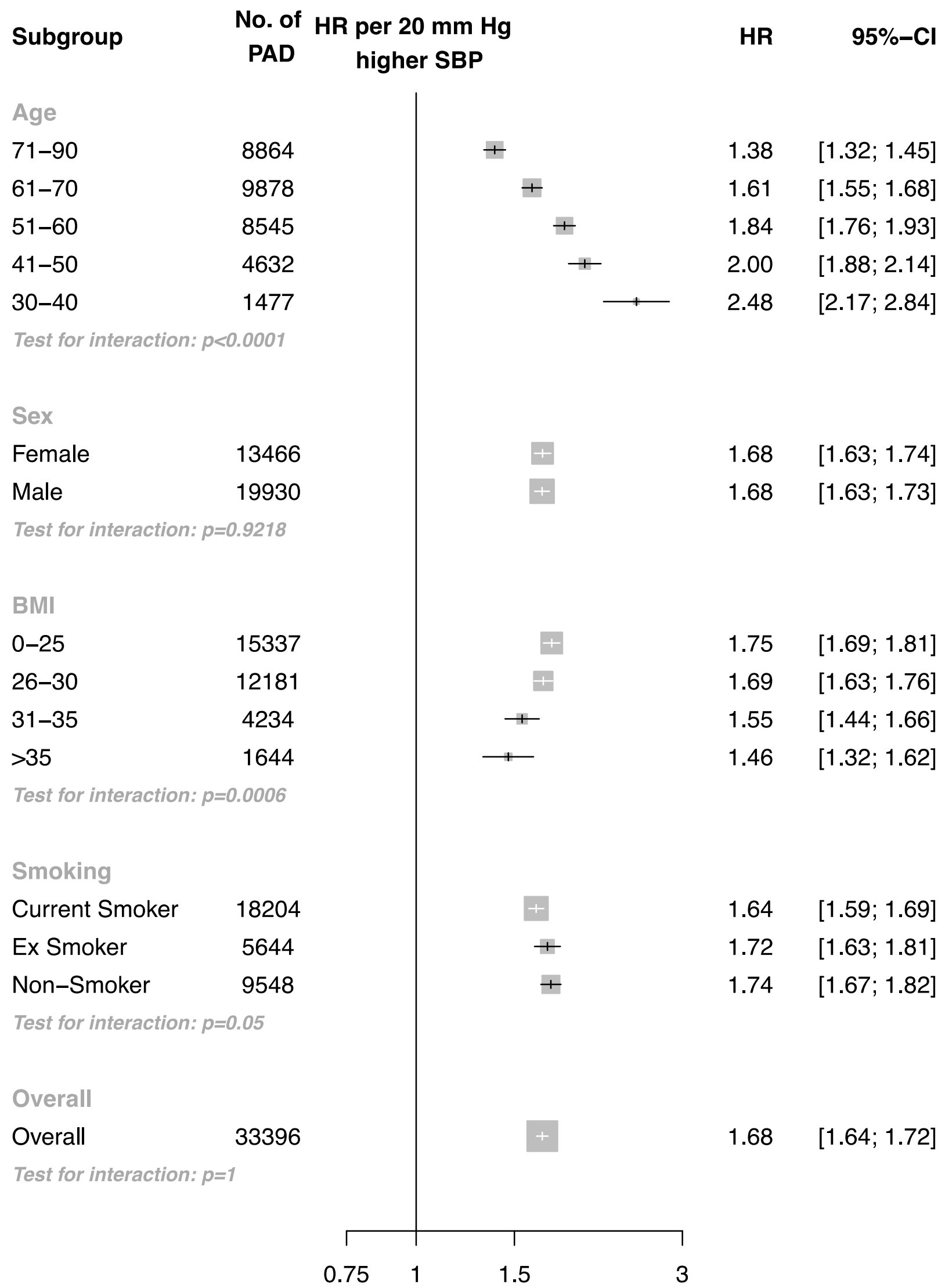

Supp. Figure 8. Adjusted hazard ratios of 20 mm Hg higher usual systolic blood pressure for incident peripheral arterial disease stratified by patient subgroup. Adjustments were for age, body mass index, smoking status, sex, baseline diabetes, baseline antihypertensive use and baseline lipid lowering drug use. For subgroups of age, adjustment was also for age category and the interaction between systolic blood pressure and age category (hazard ratio shown). For subgroups of sex, adjustment was also for the interaction between sex and systolic blood pressure (hazard ratio shown). For subgroups of body mass index, adjustments were also for body mass index category and the interaction between systolic blood pressure and body mass index category (hazard ratio shown). Area of each square is proportional to the inverse variance of the estimate. Individuals prescribed blood pressure lowering drugs at baseline are excluded.

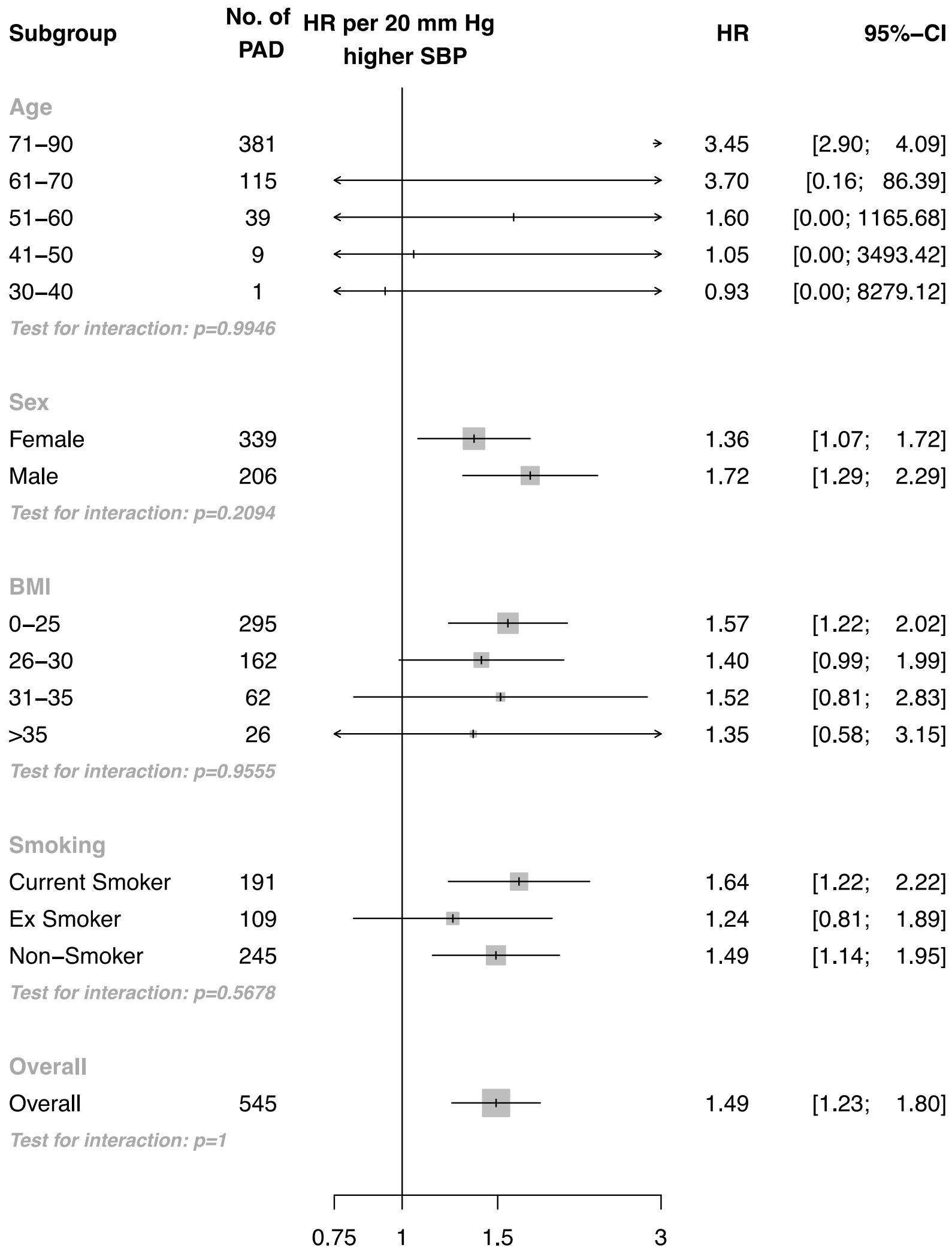

Supp. Figure 9. Adjusted hazard ratios of 20 mm Hg higher usual systolic blood pressure for incident peripheral arterial disease stratified by patient subgroup. Adjustments were for age, body mass index, smoking status, sex, baseline diabetes, baseline antihypertensive use and baseline lipid lowering drug use. For subgroups of age, adjustment was also for age category and the interaction between systolic blood pressure and age category (hazard ratio shown). For subgroups of sex, adjustment was also for the interaction between sex and systolic blood pressure hazard ratio shown). For subgroups of body mass index, adjustments were also for body mass index category and the interaction between systolic blood pressure and body mass index category (hazard ratio shown). Area of each square is proportional to the inverse variance of the estimate. Events were restricted to fatal peripheral arterial disease events and participants restricted to those eligible for linkage to cause-specific mortality.

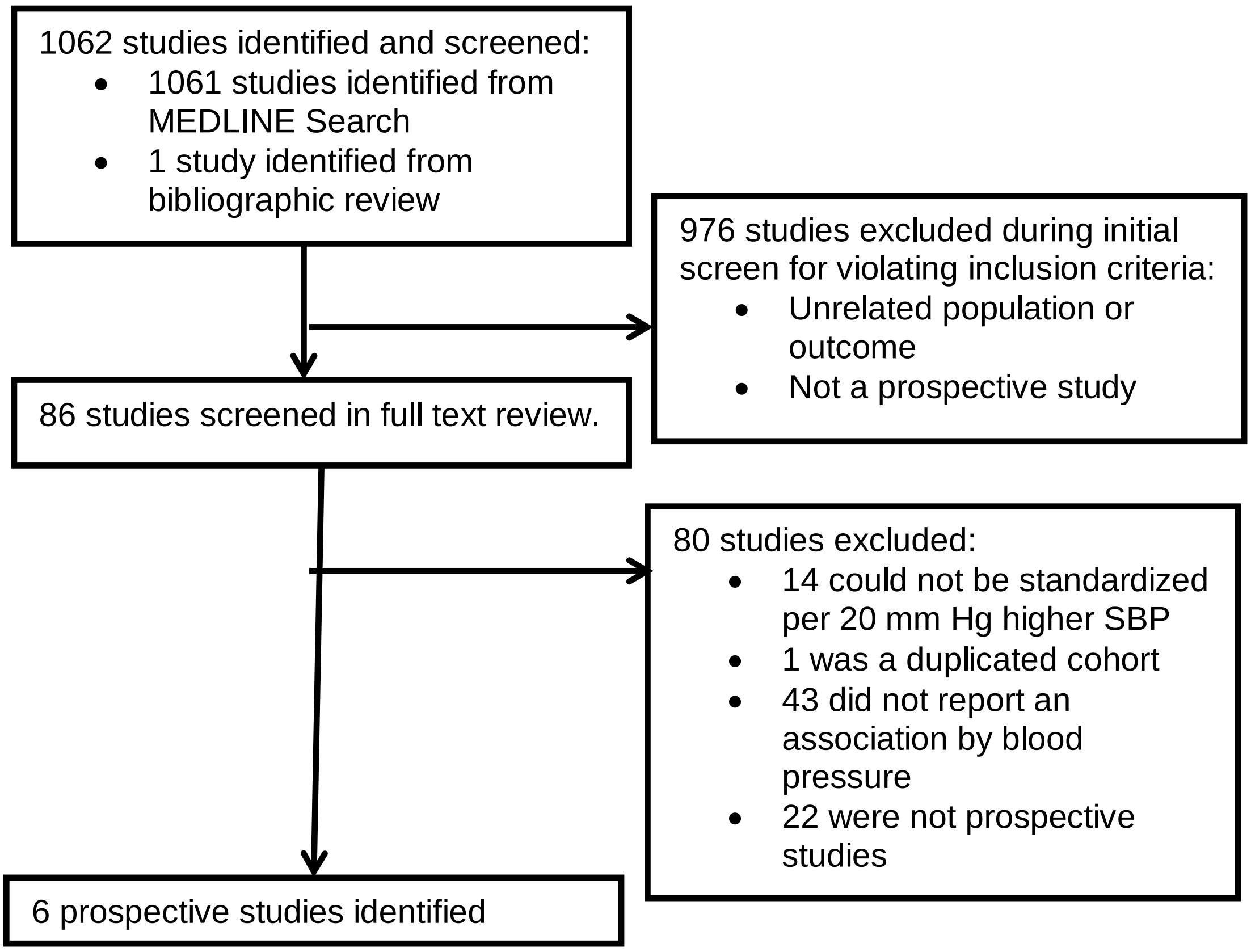

Supp. Figure 10. Identification of prospective studies which reported association of blood pressure with incident peripheral arterial disease.

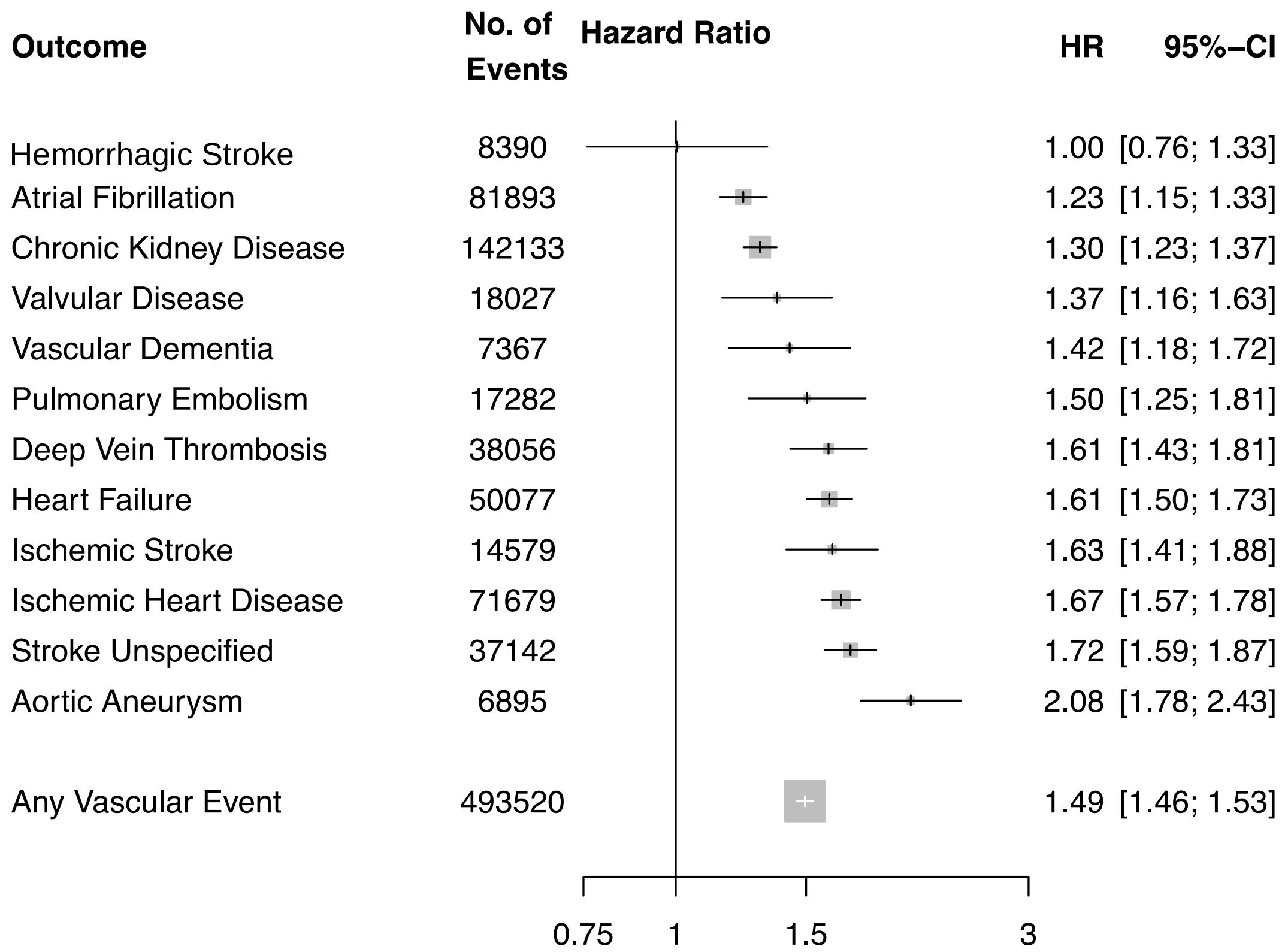

Supp. Figure 11. Adjusted hazard ratios of baseline peripheral arterial disease vs. no peripheral arterial disease for twelve different vascular events. Adjustments were for age, body mass index, smoking status, sex, baseline diabetes, baseline antihypertensive use, baseline lipid lowering drug use, total cholesterol, high density lipoprotein cholesterol and baseline peripheral arterial disease (hazard ratio shown). Area of each square is proportional to the inverse variance of the estimate.

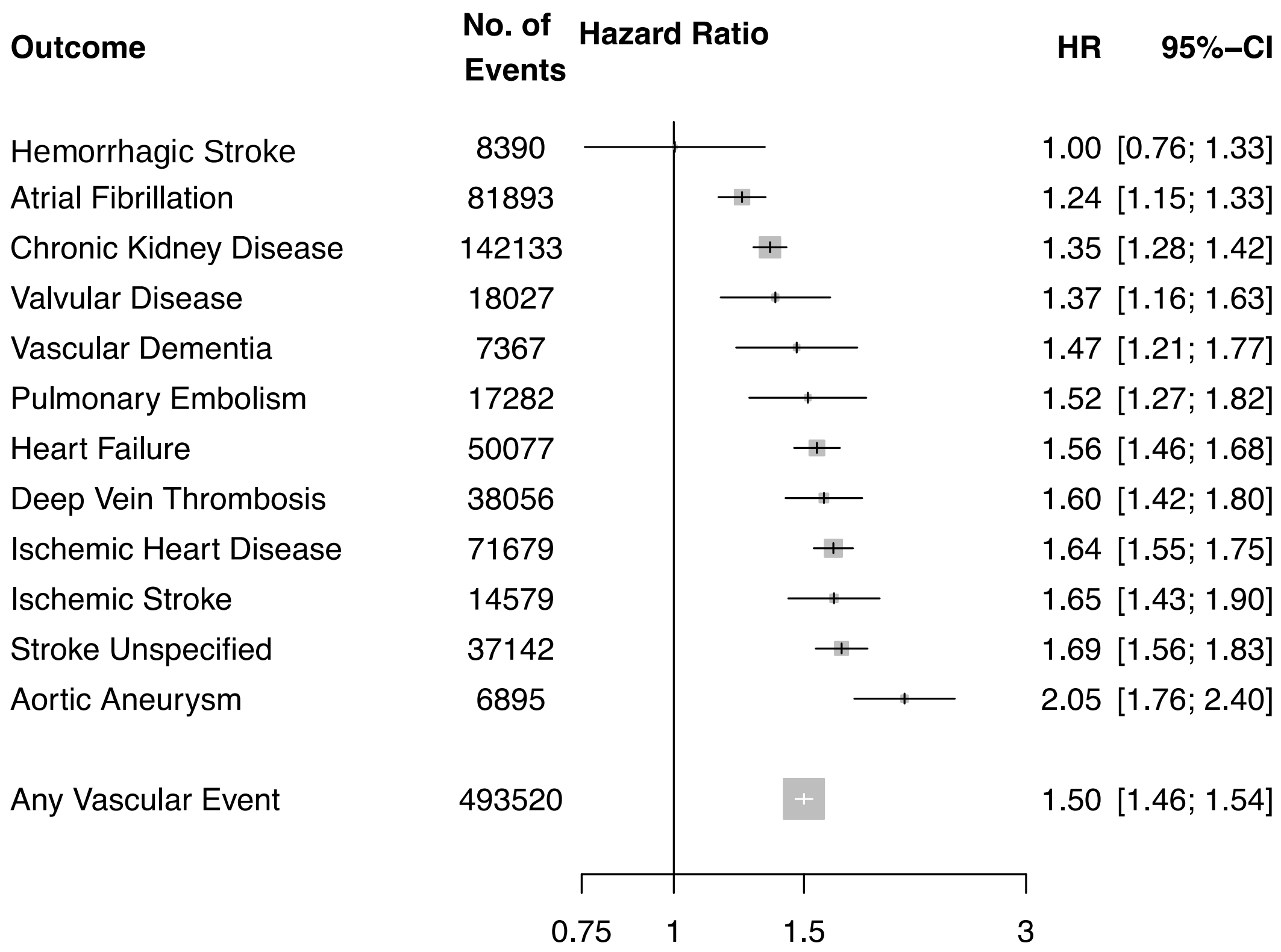

Supp. Figure 12. Adjusted hazard ratios of baseline peripheral arterial disease vs. no peripheral arterial disease for twelve different vascular events. Adjustments were for age, body mass index, smoking status, sex, baseline diabetes, baseline antihypertensive use, baseline lipid lowering drug use, total cholesterol, high density lipoprotein cholesterol, period of blood pressure measurement and baseline peripheral arterial disease (hazard ratio shown). Area of each square is proportional to the inverse variance of the estimate.

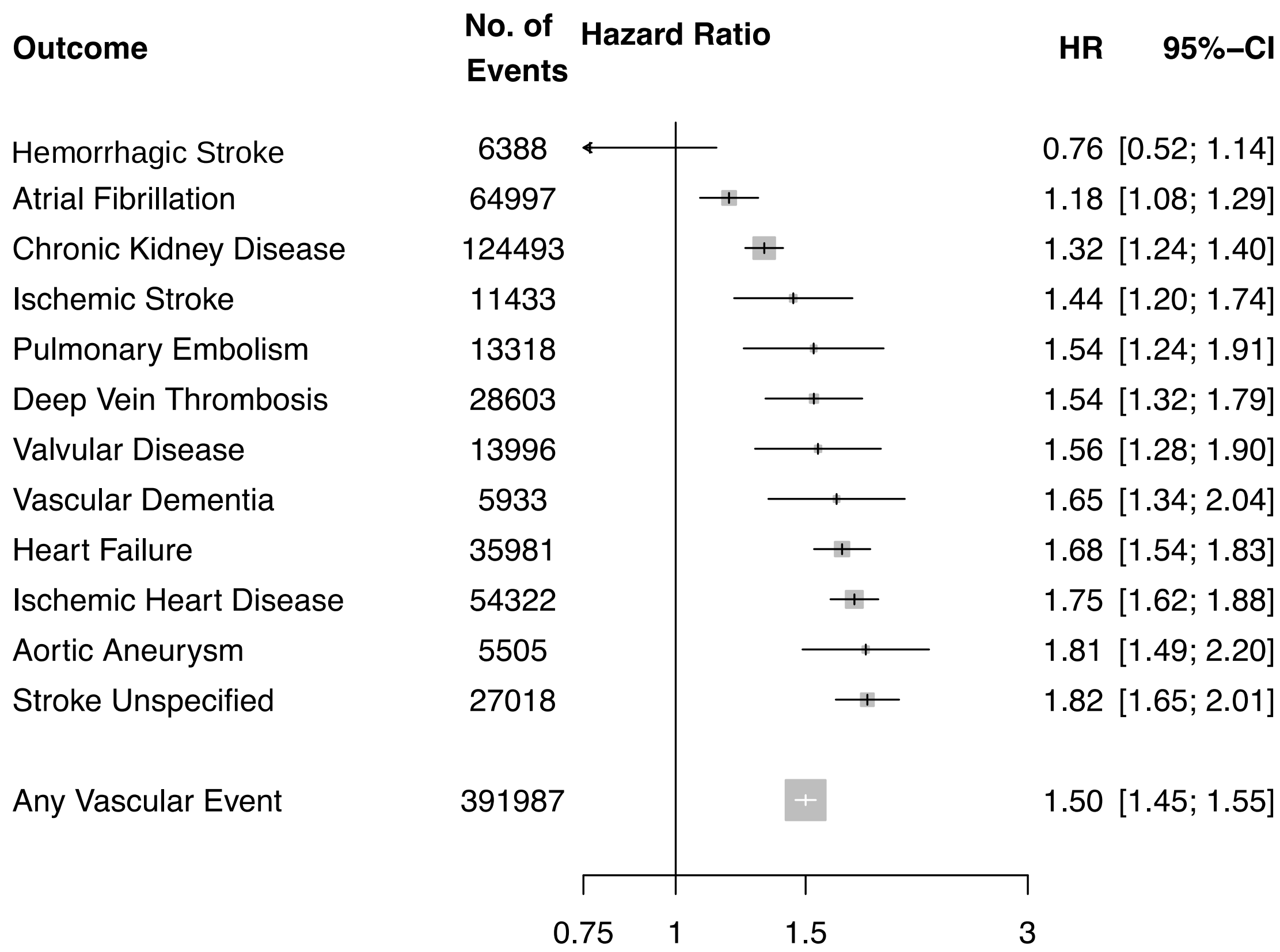

Supp. Figure 13. Adjusted hazard ratios of baseline peripheral arterial disease vs. no peripheral arterial disease for twelve different vascular events. Adjustments were for age, body mass index, smoking status, sex, baseline diabetes, baseline antihypertensive use, baseline lipid lowering drug use, and baseline peripheral arterial disease (hazard ratio shown). First two years of follow up are excluded. Area of each square is proportional to the inverse variance of the estimate.

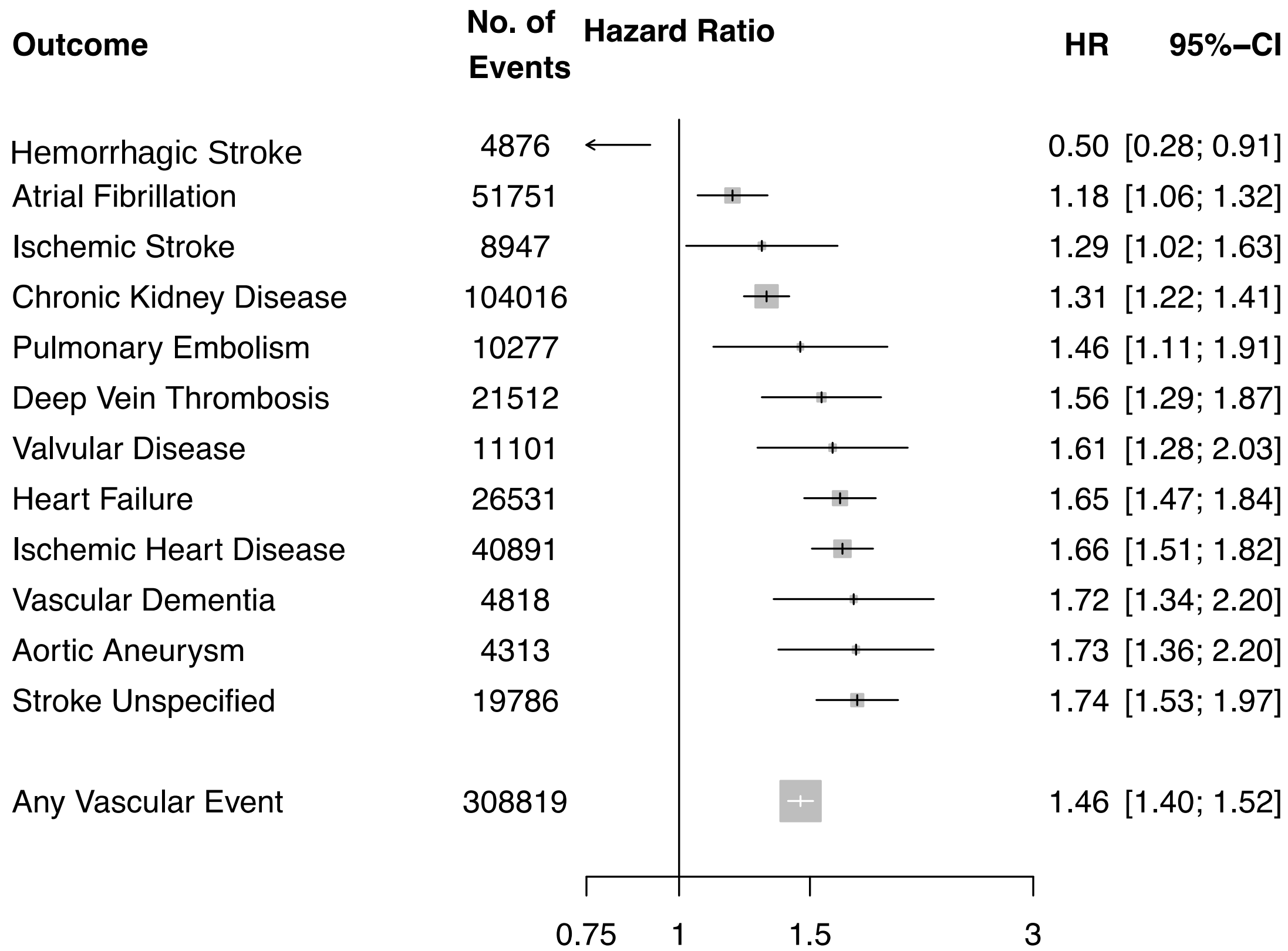

Supp. Figure 14. Adjusted hazard ratios of baseline peripheral arterial disease vs. no peripheral arterial disease for twelve different vascular events. Adjustments were for age, body mass index, smoking status, sex, baseline diabetes, baseline antihypertensive use, baseline lipid lowering drug use, and baseline peripheral arterial disease (hazard ratio shown). First four years of follow up are excluded. Area of each square is proportional to the inverse variance of the estimate.

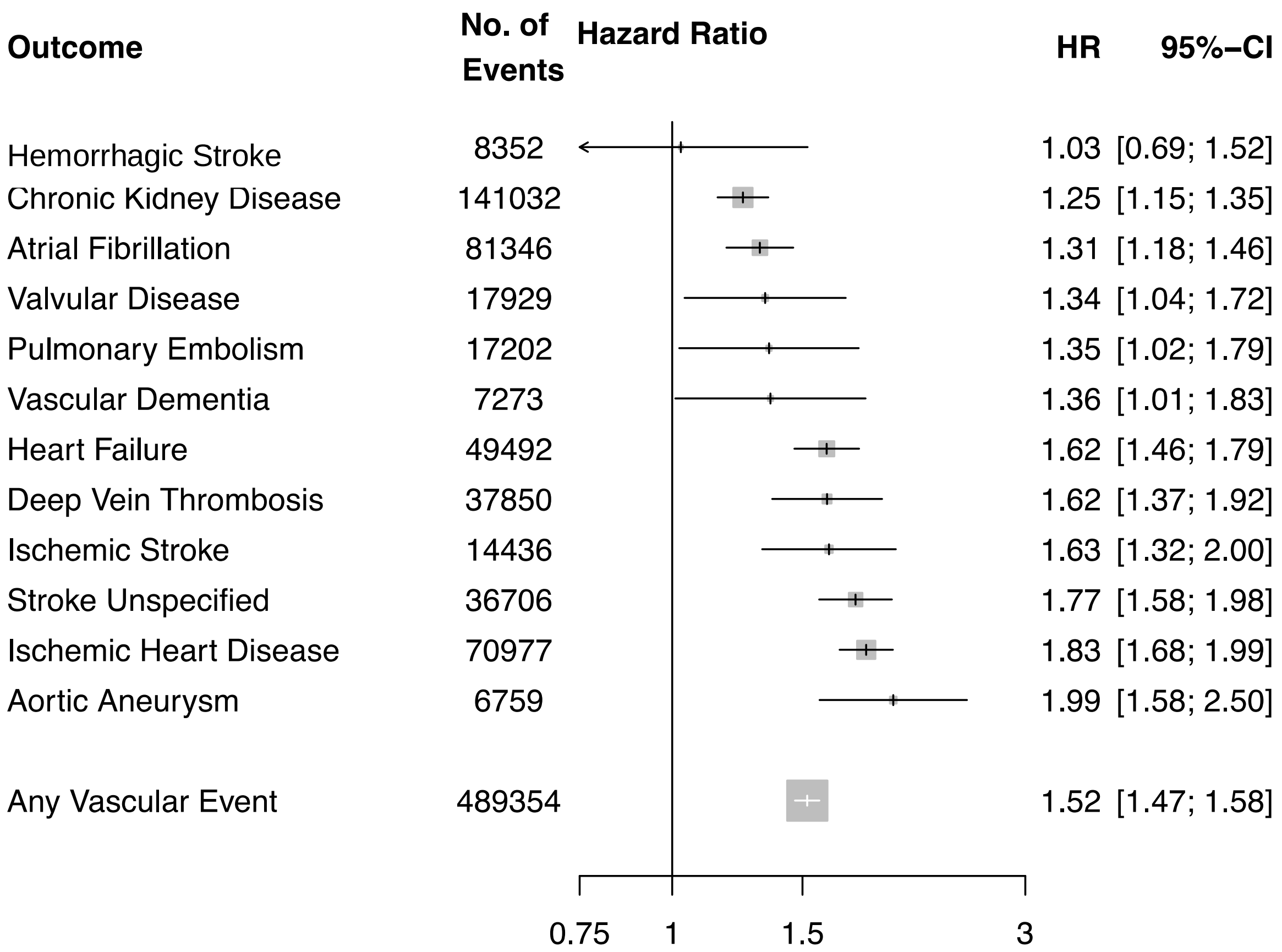

Supp. Figure 15. Adjusted hazard ratios of baseline peripheral arterial disease vs. no peripheral arterial disease for twelve different vascular events. Adjustments were for age, body mass index, smoking status, sex, baseline diabetes, baseline antihypertensive use, baseline lipid lowering drug use, and baseline peripheral arterial disease (hazard ratio shown). Peripheral arterial disease diagnosed earlier than three years prior is excluded. Area of each square is proportional to the inverse variance of the estimate.

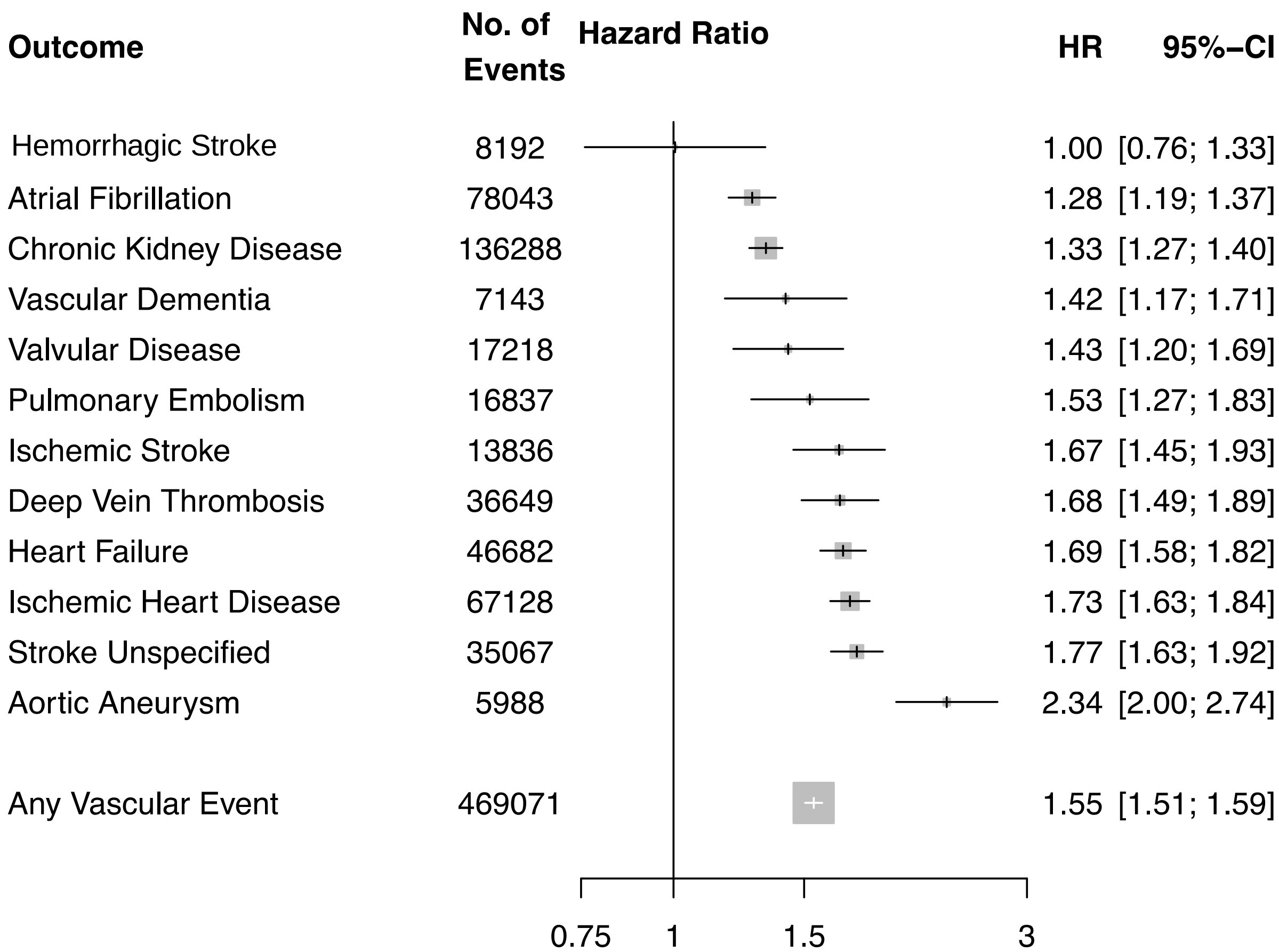

Supp. Figure 16. Adjusted hazard ratios of baseline peripheral arterial disease vs. no peripheral arterial disease for twelve different vascular events. Adjustments were for age, body mass index, smoking status, sex, baseline diabetes, baseline antihypertensive use, baseline lipid lowering drug use, and baseline peripheral arterial disease (hazard ratio shown). Individuals with diagnoses of peripheral arterial disease after baseline blood pressure measurement are excluded. Area of each square is proportional to the inverse variance of the estimate.

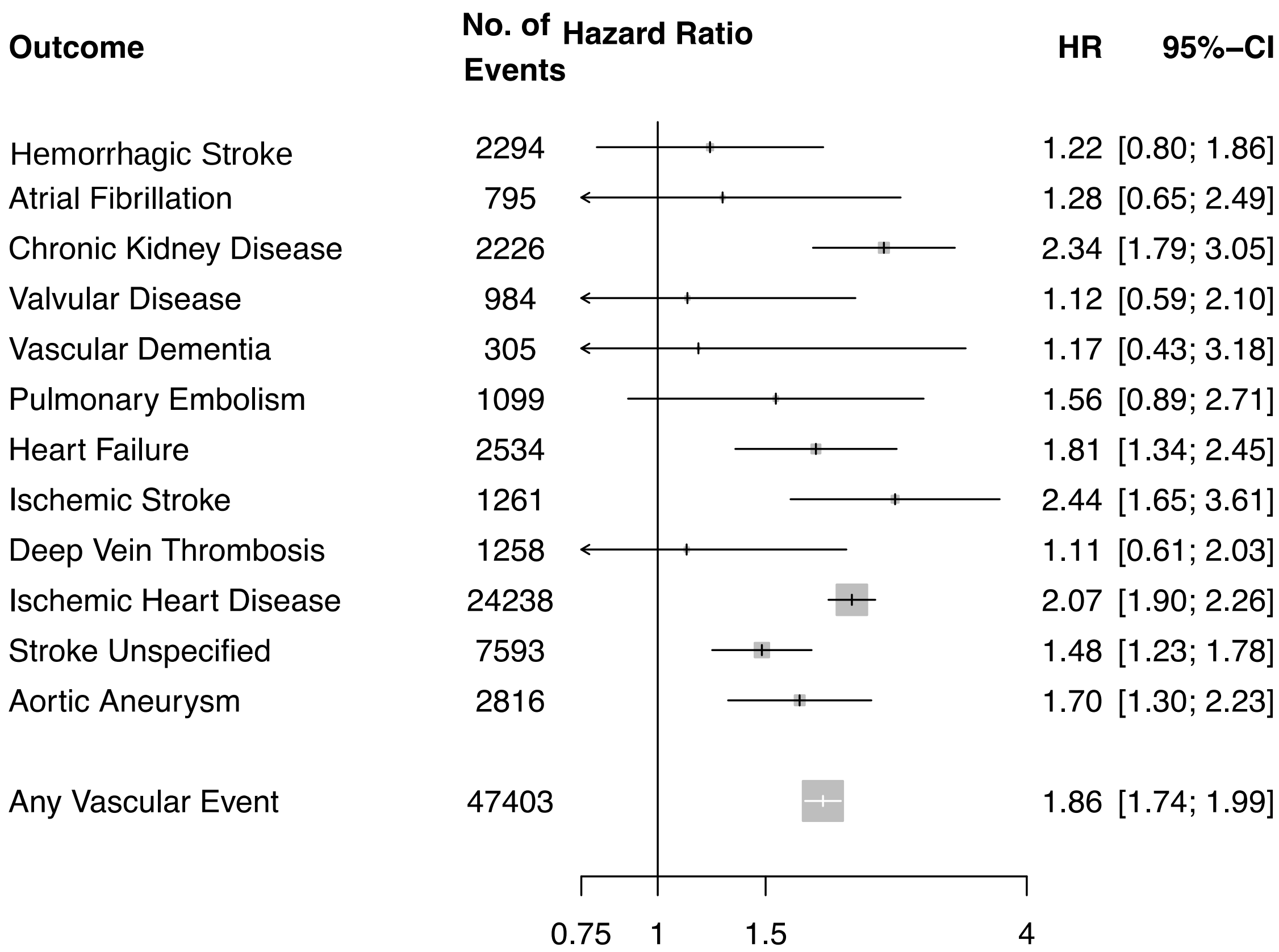

Supp. Figure 17. Adjusted hazard ratios of baseline peripheral arterial disease vs. no peripheral arterial disease for twelve different vascular events. Adjustments were for age, body mass index, smoking status, sex, baseline diabetes, baseline antihypertensive use, baseline lipid lowering drug use, and baseline peripheral arterial disease (hazard ratio shown). Area of each square is proportional to the inverse variance of the estimate. Events were restricted to fatal peripheral arterial disease events and participants restricted to those eligible for linkage to cause-specific mortality.

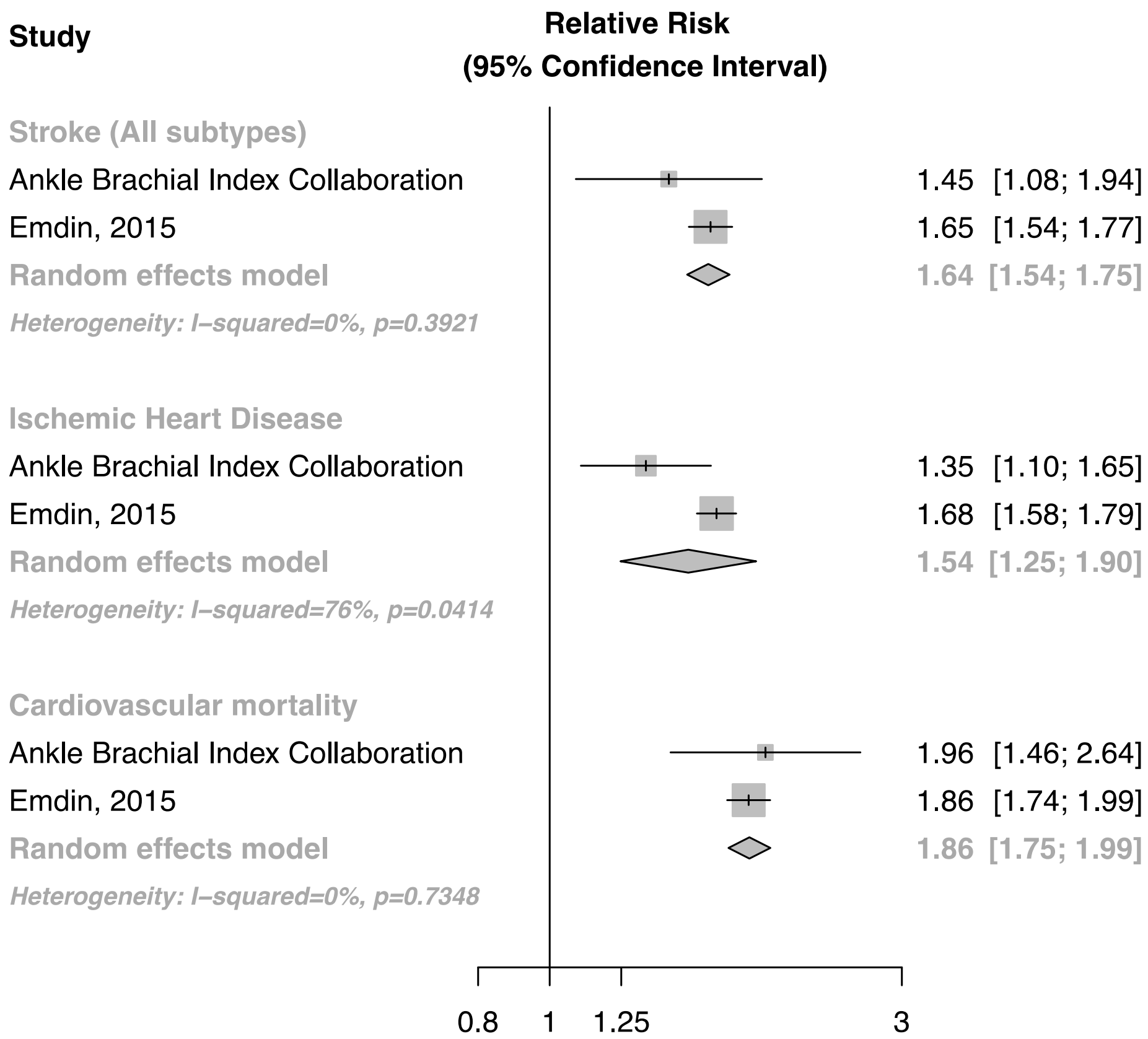

Supp. Figure 18. Pooled hazard ratios of baseline peripheral arterial disease vs. no peripheral arterial disease for vascular mortality, ischemic heart disease and stroke.

Web References

1. Rapsomaniki E, Timmis A, George J, et al. Blood pressure and incidence of twelve cardiovascular diseases: lifetime risks, healthy life-years lost, and age-specific associations in 1·25 million people. *The Lancet*. 2014;383(9932):1899-1911. doi:10.1016/S0140-6736(14)60685-1.

2. Adler AI, Stevens RJ, Neil A, Stratton IM, Boulton AJM, Holman RR. UKPDS 59: hyperglycemia and other potentially modifiable risk factors for peripheral vascular disease in type 2 diabetes. *Diabetes care*. 2002;25(5):894-899.

3. Baena-Díez JM, Bermúdez-Chillida N, García-Lareo M, et al. [Role of pulse pressure, systolic blood pressure, and diastolic blood pressure in the prediction of cardiovascular risk. Cohort study]. *Med Clin (Barc)*. 2008;130(10):361-365.

4. Baker JF, Schumacher HR, Krishnan E. Serum uric acid level and risk for peripheral arterial disease: analysis of data from the multiple risk factor intervention trial. *Angiology*. 2007;58(4):450-457. doi:10.1177/0003319707303444.

5. Cockcroft JR, Wilkinson IB, Evans M, et al. Pulse pressure predicts cardiovascular risk in patients with type 2 diabetes mellitus. *American Journal of Hypertension*. 2005;18(11):1463–7–discussion1468–9. doi:10.1016/j.amjhyper.2005.05.009.

6. Gordon T, Kannel WB. Predisposition to atherosclerosis in the head, heart, and legs. The Framingham study. *JAMA*. 1972;221(7):661-666.

7. Powell TM, Glynn RJ, Buring JE, Creager MA, Ridker PM, Pradhan AD. The relative importance of systolic versus diastolic blood pressure control and incident symptomatic peripheral artery disease in women. *Vasc Med*. 2011;16(4):239-246. doi:10.1177/1358863X11413166.

8. Shah AD, Langenberg C, Rapsomaniki E, et al. Type 2 diabetes and incidence of cardiovascular diseases: a cohort study in 1·9 million people. *Lancet Diabetes Endocrinol*. 2014;0(0). doi:10.1016/S2213-8587(14)70219-0.

9. Herrett E, Thomas SL, Schoonen WM, Smeeth L, Hall AJ. Validation and validity of diagnoses in the General Practice Research Database: a systematic review. *Br J Clin Pharmacol*. 2010;69(1):4-14. doi:10.1111/j.1365-2125.2009.03537.x.

10. Morley KI, Wallace J, Denaxas SC, et al. Defining disease phenotypes using national linked electronic health records: a case study of atrial fibrillation. Kiechl S, ed. *PLoS ONE*. 2014;9(11):e110900. doi:10.1371/journal.pone.0110900.

11. Jameson K, Jick S, Hagberg KW, Ambegaonkar B, Giles A, O'Donoghue D. Prevalence and management of chronic kidney disease in primary care patients in the UK. *Int J Clin Pract*. 2014;68(9):1110-1121. doi:10.1111/ijcp.12454.

12. Khan NF, Harrison SE, Rose PW. Validity of diagnostic coding within the General Practice Research Database: a systematic review. *Br J Gen Pract*. 2010;60(572):e128-e136. doi:10.3399/bjgp10X483562.

13. Johansson S, Wallander MA, Ruigómez A, García Rodríguez LA. Incidence of newly diagnosed heart failure in UK general practice. *Eur J Heart Fail*. 2001;3(2):225-231.

14. Imfeld P, Brauchli Pernus YB, Jick SS, Meier CR. Epidemiology, co-morbidities, and medication use of patients with Alzheimer's disease or vascular dementia in the UK. *J Alzheimers Dis*. 2013;35(3):565-573. doi:10.3233/JAD-121819.
